# Investigating Multimodal Diagnostic Eye Biomarkers of Cognitive Impairment by Measuring Vascular and Neurogenic Changes in the Retina

**DOI:** 10.3389/fphys.2018.01721

**Published:** 2018-12-06

**Authors:** Delia Cabrera DeBuc, Gabor Mark Somfai, Edmund Arthur, Maja Kostic, Susel Oropesa, Carlos Mendoza Santiesteban

**Affiliations:** ^1^Department of Ophthalmology, Bascom Palmer Eye Institute, University of Miami, Miami, FL, United States; ^2^Retinology Unit, Pallas Kliniken, Olten, Switzerland; ^3^Department of Ophthalmology, Semmelweis University, Budapest, Hungary

**Keywords:** eye biomarkers, retinal vascular complexity, Alzheimer’s disease, cognitive impairment, fractal dimension, neurodegeneration, electroretinography

## Abstract

Previous studies have demonstrated that cognitive impairment (CI) is not limited to the brain but also affects the retina. In this pilot study, we investigated the correlation between the retinal vascular complexity and neurodegenerative changes in patients with CI using a low-cost multimodal approach. Quantification of the retinal structure and function were conducted for every subject (*n* = 69) using advanced retinal imaging, full-field electroretinogram (ERG) and visual performance exams. The retinal vascular parameters were calculated using the Singapore Institute Vessel Assessment software. The Montreal Cognitive Assessment was used to measure CI. Pearson product moment correlation was performed between variables. Of the 69 participants, 32 had CI (46%). We found significantly altered microvascular network in individuals with CI (larger venular-asymmetry factor: 0.7 ± 0.2) compared with controls (0.6 ± 0.2). The vascular fractal dimension was lower in individuals with CI (capacity, information and correlation dimensions: D_0_, D_1,_ and D_2_ (mean ± SD): 1.57 ± 0.06; 1.56 ± 0.06; 1.55 ± 0.06; age 81 ± 6years) vs. controls (1.61 ± 0.03; 1.59 ± 0.03; 1.58 ± 0.03; age: 80 ± 7 years). Also, drusen-like regions in the peripheral retina along with pigment dispersion were noted in subjects with mild CI. Functional loss in color vision as well as smaller ERG amplitudes and larger peak times were observed in the subjects with CI. Pearson product moment correlation showed significant associations between the vascular parameters (artery-vein ratio, total length-diameter ratio, D_0_, D_1_, D_2_ and the implicit time (IT) of the flicker response but these associations were not significant in the partial correlations. This study illustrates that there are multimodal retinal markers that may be sensitive to CI decline, and adds to the evidence that there is a statistical trend pointing to the correlation between retinal neuronal dysfunction and microvasculature changes suggesting that retinal geometric vascular and functional parameters might be associated with physiological changes in the retina due to CI. We suspect our analysis of combined structural-functional parameters, instead of individual biomarkers, may provide a useful clinical marker of CI that could also provide increased sensitivity and specificity for the differential diagnosis of CI. However, because of our study sample was small, the full extent of clinical applicability of our approach is provocative and still to be determined.

## Introduction

According to the 2015 World Alzheimer Report, there are approximately 46 million dementia patients worldwide (Prince et al., 2015). This number will almost double every 20 years, and it is estimated to increase to 131.5 million by 2050. It has been estimated that the total worldwide dementia-related healthcare cost is $818 billion, rising to $2 trillion by 2030 (Prince et al., 2015).

Alzheimer’s disease (AD) is the most common, progressive cause of dementia in the elderly, and a severe burden on the aging society worldwide (Prince et al., 2015). Also, dementia is most common among older patients with longer Parkinson’s disease (PD) duration, and least common in individuals with multiple sclerosis (MS). Previous studies have suggested that AD initiates decades before it is clinically expressed (La Rue and Jarvik, 1987; Linn et al., 1995; Snowdon et al., 1996; Braak and Braak, 1997; Elias et al., 2000; Kawas et al., 2003). Therefore, it would be possible to identify persons who will ultimately express the disorder long before the early symptoms appear as well as to target potential interventions to prevent disease expression in such individuals at high risk.

As an anatomically integral part of the brain, the retina shares important structural and pathogenic pathways with the central nervous system (Cabrera DeBuc et al., 2017). The link between eye pathology and AD, PD and MS has been established in multiple studies (Katz and Rimmer, 1989; London et al., 2012; Cabrera DeBuc et al., 2017; Hampel et al., 2018). In particular, neuronal loss in AD associated with optic nerve parameters include retinal ganglion cells which are similar to neurons in the cerebral cortex, and have been correlated to neurodegeneration in AD (Katz and Rimmer, 1989; Hampel et al., 2018). Recently, advances in neuro-electrophysiological and optical imaging technologies have facilitated non-invasive morphological and functional measurements in the eye using electroretinography and advanced retinal imaging. Specifically, retinal microvascular changes as well as the abnormal bioelectrical activity of retinal ganglion cells, photoreceptors and the optic nerve have been associated with cognitive decline and brain changes in relation to aging and early AD (Moschos et al., 2012; Ong et al., 2014; Hampel et al., 2018). Moreover, it has been hypothesized that if an association can be made between the amyloid in the brain and the amyloid in the eye, then it would be feasible to diagnose AD by looking into the eye (Koronyo-Hamaoui et al., 2011; Koronyo et al., 2012; Hampel et al., 2018). Therefore, the vast research exploring cognitive impairment non-invasively in the brain through the easily accessible retina warrants further investigation to support the use of retinal biomarkers in the detection of cognitive decline even during the asymptomatic period.

The discovery of biomarkers is a complicated process that demands considering multiple factors and approaches to obtain reliable markers that allow us to predict risk or response to treatment very early and with low false positive and false negative rates. Unfortunately, the critical barriers to primary prevention of cognitive decline are the lack of rapid, non-invasive, sensitive and low-cost biomarkers. In this pilot study, we investigated the extent to which measures of vascular complexity and neurodegenerative changes in the retinal tissue contribute to differences in cognitive function using a low-cost multimodal approach. Our central hypothesis is that multivariate eye biomarkers reflect distinctive eye-brain signatures of cognitive impairment that might be associated with the onset and progression of cognitive decline. Therefore, quantification of the retinal vascular network complexity and its neural function was performed for each study participant using advanced retinal imaging, full-field electroretinogram (ERG) as well as visual performance exams. Our preliminary findings show that our multimodal approach to evaluating visual capacities in elderly individuals may add predictive value of early visual pathway injury associated with cognitive decline and facilitate the introduction of novel multimodal eye biomarkers for early detection of cognitive impairment at a low-cost.

## Materials and Methods

The Human Research Ethics Committee of the University of Miami, Miami, FL, United States approved all protocols and methods described in this study. The research adhered to the tenets outlined in the Declaration of Helsinki. Informed consent was obtained from all participants following a thorough explanation of all test procedures. All study subjects underwent cognitive function assessment and ERG followed by advanced retinal imaging, color vision test and visual performance exams of both eyes.

### Study Participants

Prospective subjects with cognitive impairment were identified in a non-systematic fashion as they appeared in the clinic or identified from a population attending adult care centers and community clinics with a diagnosis of AD. Study subjects were recruited in numerous ways using flyers, a university press release that generated interest in the community of Miami-Dade and Broward counties in Florida, and by giving talks to AD caregiver support groups in the nearby regions. Study subjects (or for the patients with cognitive decline, reliable caregivers/informants) were interrogated about subjective changes in vision that may have occurred in the recent past or over the progression of their disease. The exclusion criteria were age under 55 years and the presence of any ophthalmic history before recruitment. Participants who were not capable of comprehending information, and making decisions about participation in the study due to cognitive impairments that affect decision-making to make informed choices, had informed consent obtained through a proxy. The macular and optic disk regions were scrutinized for abnormalities and subjects without any ocular history except for cataract surgery were included in the analyses. All subjects wore their own best optical correction for the visual performance tests. Both hypertension and diabetes mellitus as well as cardiovascular disease were considered comorbid medical conditions related to retinal vascular alterations. Also, current or history of study subject-reported smoking categorized as current, past, or never, was considered because of earlier reports linking smoking with potential vascular changes in the retina (Sun et al., 2009).

### Fundus Imaging and Quantitative Analysis of the Retinal Vascular Network

**FIGURE 1:**
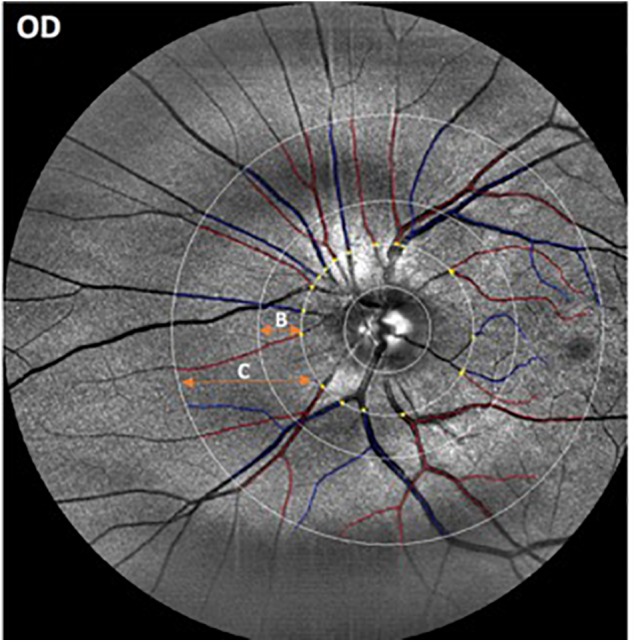
Representative image obtained with the EasyScan unit (i-Optics Corporation, Netherlands) and analyzed with the SIVA program that measured the caliber of the vessels emerging from the optic disk. Arterioles are in red and venules are in blue. The SIVA software automatically detects the optic disk and traces vessels in a zone 0.5 to 2.0-disk diameter from the disk margin. The different circular ROIs with various radii around the optic disk center are labeled as B (0.5 – 1.0 disk diameters away from the disk margin) and C (0.5- 2.0 disk diameters away from the disk).

Retinal fundus photographs were taken of each eye with a non-mydriatic digital camera (EasyScan, iOptics, Netherlands) based on scanning laser ophthalmoscopy (SLO) technology that has better penetration of media opacities such as cataract (Webb et al., 1987). Its high-resolution images reveal what cannot be seen with a traditional fundus camera, thanks to its multiple plane principle (Figure 1). This low-cost camera with a FOV of 45° and image size of 1024 × 1024 requires minimal operator training, and it is conceived to maximize patient flow. Also, its compact, ergonomic design and low power flash help ensure patient comfort. Moreover, taking a high-contrast, detailed retinal image is easy and intuitive. With one push of a button, it can be operated anywhere and captures the image in both eyes in less than 5 min.

Retinal images from all participants were masked and collected for further analysis. The right eye was imaged first followed by the left eye. Optic-disk centered images of a selected eye from each participant were analyzed with a semi-automated computer-assisted program, Singapore I Vessel Assessment (software version 3.0, National University of Singapore) (Cheung et al., 2011). Images with poor quality were removed from the analysis. The investigation was performed using a standardized protocol by a trained grader after the retinal arterioles and venules were identified automatically by the SIVA program (Figure 1). The circular retinal region of interest (ROI) for the overall analyses were 0.5 – 1.0 disk diameters away from the disk margin (zone B in Figure 1) or 0.5–2.0 disk diameters away from the disk margin (zone C in Figure 1). This particular ROI selection to measuring the geometric vascular parameters warranted that the retinal vessels had reached arteriolar status. All artifacts traced as vessels were removed by comparing the automated vessel tracing with the fundus images obtained with the EasyScan unit. Two experienced retinal specialists revised the vessel classification (i.e., arteries/veins) automatically generated by the SIVA software, and assessed all fundus photographs to identify and rule out retinal pathological features related to age-related macular degeneration (AMD), diabetic retinopathy and glaucoma. Then, all misclassifications of the retinal vessels were corrected by the grader, and images showing signs of AMD and other pathological features related to diabetic retinopathy (e.g., exudates, edema, cotton wool spots, hemorrhages, microaneurysms) and glaucoma (suspicious optic disk cupping) were discarded. Moreover, the resulting geometric vascular parameters of the retina were obtained and used for further analysis: retinal vascular caliber, summarized as central retinal artery/vein equivalent (CRAE, CRVE), curvature tortuosity (cTORTa, cTORTv), branching coefficient (BCa, BCv), branching asymmetry factor (AFa, AFv), length diameter ratio (LDRa, LDRv), and artery-vein ratio (AVR) as described in earlier studies (Cheung et al., 2011). The reliability assessment and detailed characterization of these vascular parameters have been described elsewhere (Liew et al., 2008; Cheung et al., 2011). The SIVA program calculates the CRAE and CRVE parameters, based on the revised Knudtson–Parr–Hubbard formula. These parameters represent the average width of the central retinal vessels. The AVR consists of a ratio of the caliber of arterioles to venules, and it is not affected by magnification differences caused by refractive errors and camera lens adjustments (Cosatto et al., 2010). The BC is an estimate of the ratio between the diameters of the main vessel and the diameters of its branches, which is also known as daughter vessels (Zamir et al., 1979). Therefore, a vascular network with comparably sized vessel diameters between the main vessel and its branch is characterized by a higher BC, while a reduction in the branches’ diameters compared to the main vessel is related to a lower BC. Also, cTORT is a tortuosity index defined as the integral of curvature squared along the path of the vessel normalized by the total arc length (Hart et al., 1999). Therefore, cTORT considers the bowing and points of inflection. Straighter vessels are characterized by a lower tortuosity index. The LDR is a measure of the vessel width defined as the length of the vessel from the midpoint of one bifurcation to the midpoint of the next bifurcation. It is expressed as a ratio to the diameter of the parent vessel at the first bifurcation (Cheung et al., 2011). The ratio of the squares of the two branching vessel widths is used to calculate AFa and AFv.

#### Fractal Dimension of the Retinal Vascular Network

Fractal analysis, a mathematical method used to measure complexity in natural phenomenon (Mandelbrot, 1982), is a well proved and reliable methodology used to characterize the retinal vasculature (Liew et al., 2008; Cosatto et al., 2010; Thomas et al., 2014). This method was introduced in ophthalmology by Family et al. (1989), and since then, interest in investigating the association between the fractal dimension (FD) of the retinal vasculature and disease severity and progression has dramatically increased. The retinal vasculature tree could be quantified with various methods of fractal analysis (Stosic and Stosic, 2006; Macgillivray et al., 2007; Ţălu, 2013a,b). The vascular FD, characterizes a “global” measure that includes the whole branching pattern of the retinal vascular tree. Therefore, a more complex branching pattern indicates a larger FD value. Self-similarity over different scales is an important property of the fractal structures. This self-similar property means that at different magnifications or scales, a similar pattern with different sizes can be perceived. This property can be described by the following equation:

(1) $$N(r)=\mathrm{const}\;r^{-D}$$

where N(r) is certain measurements applied on the complex pattern of the fractal structure at a scale or magnification r; D is the FD that implies how many new similar patterns are observed as the resolution magnification (scale) increases or decreases.

**FIGURE 2:**
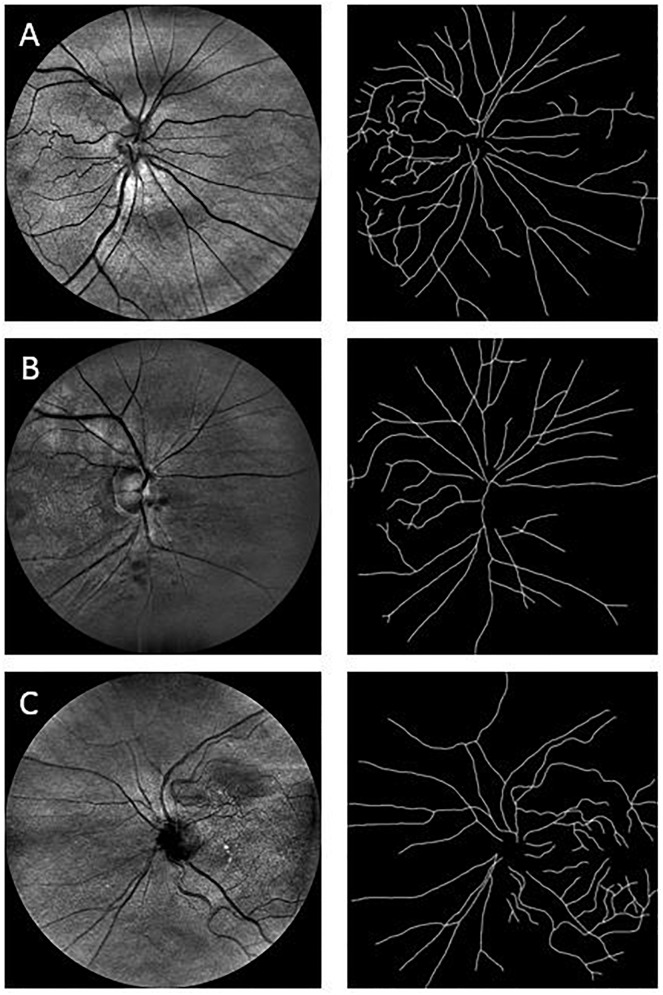
Sample images used in the fractal analysis. Images in the left column are the raw images obtained with the EasyScan system, while those in the right are their respective skeleton images that were used in the fractal analysis. Row **(A)** is from a healthy cognitively individual (MoCA score range: 29.6–25.2), Row **(B)** is from an MCI subject (MoCA score range: 25.2–19), and Row **(C)** is from a participant with more cognitive deterioration than MCI (MoCA score range: 21 to 11.4). MoCA, Montreal Cognitive Assessment.

Because the human retinal vessel structures have been shown to be geometrical multifractals (Family et al., 1989; Mainster, 1990; Kyriacos et al., 1997; Stosic and Stosic, 2006; Ţălu, 2013b), the vascular FD was calculated from the skeletonized vascular network (Figure 2) using both a monofractal and multifractal approach (Vehel and Legrand, 2003). In contrast to most studies, our approach did not use different circular regions of interest with various radii around the optic disk centers. Instead, to obtain comparable FD values, the skeleton comprised the whole branching pattern observable in the full 45° FOV.

The box-counting method, proposed by Liebovitch and Toth (1989), is the most popular monofractal approach for estimating the FD of fractal objects. This method generates data by covering the object with a rectangular coordinate grid and breaking the data into boxes and then analyzing the subsets by counting the number of boxes. Therefore, the measurement N(r) in (1) is the number of boxes with side-length r that overlap with the vessel segmentation or skeleton, and the box dimension (D_B_) can be calculated as the absolute value of the slope of N(r) plotted against r in a log-log plot.

The multifractal approach was used to investigate the effect of the scale on the multifractal dimension (Stosic and Stosic, 2006; Gould et al., 2011). In this approach, the multifractal behavior in the structure is described by finding the generalized dimension Dq, which is associated to a value of q that expresses the fractal properties in different scales. The plot of Dq vs. q is usually sigmoidal and decreasing to a multifractal configuration. This method has been employed effectively to prove geometric multifractality of the diffusion limited aggregation (DLA) fractal dimension (Vicsek et al., 1990). Certain studies consider determined values of Dq (e.g., D_0_, D_1,_ and D_2_), which describe the multifractal characteristics of an object when condition D_0_ ≥ D_1_ ≥ D_2_ is satisfied. Particularly, the capacity dimension D_o_ (or box counting), has been reported to be constantly larger than the information (or Shannon or entropy) dimension D_1_, which was in turn always larger than the correlation dimension D_2_ (i.e., all satisfying D_o_ > D_1_ > D_2_). In all studies, all the three generalized dimensions (D_o_, D_1,_ and D_2_) are being significantly lower than the DLA fractal dimension (D ≈ 1.7) (Witten and Sander, 1981; Family et al., 1989; Mainster, 1990; Kyriacos et al., 1997; Stosic and Stosic, 2006). The D_1_ measures the uncertainty or entropy of a random event, being lower or less informative for events that happen very often while larger or more informative for events that might happen less likely (Stosic and Stosic, 2006). The D_2_ estimates the FD via the association between two pixels inside a region (Stosic and Stosic, 2006). Therefore, in our study, the multifractal behavior in the retinal images was analyzed using the generalized dimension spectrum for *q* values ranging between -10 and +10, where all dimensions were statistically examined. Accordingly, D_o,_ D_1,_ and D_2_ were computed and compared to check for consistency where D_o_ > D_1_ > D_2_.

**FIGURE 3:**
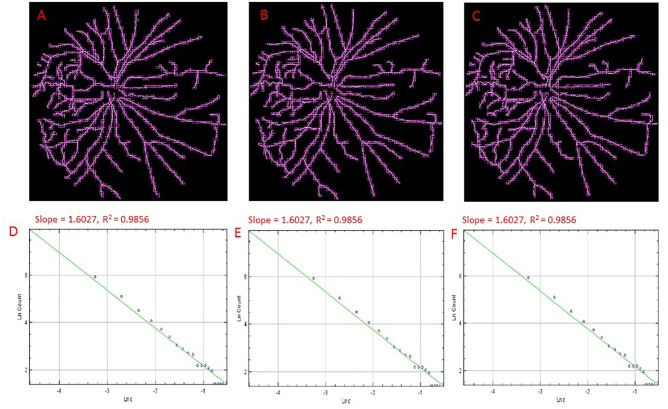
Skeletons of vessels obtained from an cSLO image of a cognitively healthy individual with overlaid boxes at different number (i.e., 9, 12, and15) of grid positions, 9 **(A)**, 12 **(B)**, and 15 **(C)** with their corresponding double-log plots **(D–F)** of the count of boxes containing meaningful pixels vs. box size showing the slope (D_0_) and the *R*^2^ values of the regression lines. Overlaid boxes on the skeletonized images are shown to indicate that only boxes containing meaningful pixels were counted and used in the computation of the fractal dimension (D_0_ or the slope). Different number of grid positions are shown in **(A–C)** from the FracLac settings to show that these parameters were optimized and that a change in the number of grid positions did not result in a change in the slope (D_0_) or the *R*^2^ values **(D–F)**. Hence the recommended number of 12 grid positions was used.

**FIGURE 4:**
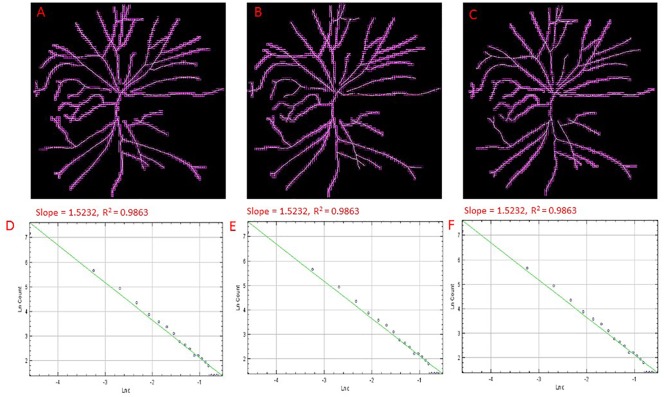
Skeletons of vessels from an cSLO image of a MCI participant with overlaid boxes at different number of grid positions, 9 **(A)**, 12 **(B)**, and 15 **(C)** with their corresponding double-log plots **(D–F)** of the count of boxes containing meaningful pixels vs. box size showing the slope (D_0_) and the *R*^2^ values of the regression lines. Overlaid boxes on the skeletonized images are shown to indicate that only boxes containing meaningful pixels were counted and used in the computation of the fractal dimension (D_0_ or the slope). Different number of grid positions are shown in **(A–C)** from the FracLac settings to show that these parameters were optimized and that a change in the number of grid positions did not result in a change in the slope (D_0_) or the *R*^2^ values **(D–F)**. Hence the recommended number of 12 grid positions was used. For all double log pots of the remaining cognitively impaired and healthy controls, see Supplementary Information.

The public domain Java image-processing program ImageJ together with the FracLac plug-in was used to calculate the multifractal properties of the retinal vasculature network (Karperien, 1993). A total of 12 different grid positions was defined in the grid design pane of the FracLac environment. This arrangement facilitated multiple scans by changing the starting position of the sampling grid each time to capture the variation attributable to the grid orientations or positions. The recommend setting for this parameter is 4 – 12 grid positions or orientations as sampling tends to be unaffected beyond 12 positions. We optimized this parameter by using 3 different number of grid positions (9, 12, and 15) to test whether the different number of grid positions may result in different slopes (D_0_) and R^2^ values in the double log plots. As shown in Figures 3, 4, the slopes and *R*^2^ values remained the same. Hence, we used the recommended number of 12 grid positions. A linear scaling method was also used to set 20 varying box sizes from a minimum box size of 10 pixels to a maximum box size of approximately 60% of the image size. The FracLac software computes the constant linear scale as the difference between the minimum and maximum box sizes divided by the number of different box sizes. The “greater dim” and “check pix” check-boxes were selected to make sure the longer side of the bounding box was used as the image dimension and that only boxes containing meaningful pixels were used in the computations of FD, respectively. No sub sampling was selected in the sub scan options. The generalized dimension spectrum was set from -10 to 10 with an increment of 1 and a graph of Dq vs. q was chosen in the MF (multifractal) Graphs options. In the data processing option, we selected “show optimal sample only” for the multifractal optimizer option and “no filter” for the multifractal filters. Regression for the double log plots and “draw grids” (to show whether grids used in the FD calculation contained meaningful pixels) options were selected in the graphics option.

### Electroretinography

Several ERG changes have been recorded in patients with AD (Sadun et al., 1987; Armstrong, 1996). Earlier studies have reported that the involvement of the visual cortex may be the cause for dysfunction of the elementary visual sensation that may be involved in the development of visual cognitive deficits and vision-related behavioral symptoms (Strenn et al., 1991; Granholm et al., 2003). Moreover, flash ERG was used to demonstrate dysfunction of the retina under photopic and scotopic conditions in patients with dementia with Lewy bodies (Devos et al., 2005). The use of the full-field ERG was also suggested to find whether dysfunction of preganglionic elements may also occur in AD (Parisi et al., 2001). Intriguingly, it has been also suggested that the ERG could be possibly used as a marker of central dopamine and serotonin levels (Lavoie et al., 2014).

Evaluation of the bioelectrical activity of the retina was performed with a full-field ERG (RETeval^TM^, LKC Technologies, Inc., Gaithersburg, MD, United States) according to the International Society for Clinical Electrophysiology of Vision (ISCEV) protocol (Marmor et al., 2004; Holder et al., 2007; Hood et al., 2008). The RETeval^TM^ system is a full-field flicker ERG recording device designed as a low-cost handheld alternative to traditional ERG screening without the need for mydriasis (Kato et al., 2015). It can perform measurements in both eyes in about 3 min without any eye contact. Also, various flicker-based or single-flash based protocols are available through a protocol chooser that enables other ERG/VEP tests (Sadun et al., 1987). The intensity of the flash source of this device is calibrated consistently with the light-adapted 3.0 flicker ERG protocol of the ISCEV standard. The ERG examination was performed by an experienced examiner trained in the use of the RETeval^TM^ unit. As per the manufacturer’s recommendations, a disposable, self-adhering skin contact electrode array (Sensor Strip; LKC Technologies) was placed on the cheek inferior to the lateral half of the lower eyelid (∼2 mm from the eyelid margin) and the lead was connected to this strip to initiate the ERG recordings. Participants were seated in an upright position and with the fellow eye covered were asked to focus on the red beam projected from the device. The right eye was tested first followed by the left eye. The skin contact electrode strips were disposed of to prevent rescreening of other study subjects using the used strips. ERG amplitudes and implicit time values were measured consistent with the recommendations by the ISCEV (McCulloch et al., 2015). The protocol used was the ISCEV 6 step, light-adapted first. Assessments consisted of light-adapted ERG (stimulus strength, 3.0 cd⋅s/m^2^; frequency, 28.3 Hz flicker response); and dark-adapted ERG including rod, maximal dark-adapted and cone responses. Implicit times and amplitude values of the ERGs elicited by 141 to 424 flashes were processed separately for each eye.

Also, all comparisons were established by using our ERG data collected along with the reference data provided by the manufacturer of the RETeval^TM^ device. Specifically, the ERG norm in the RETeval^TM^ system is based on reference data collected from 244 individuals aged 4–85 who were carefully examined to have normal vision. The criteria followed to classify the eyes as normal were a BCVA of 20/25 (0.1 logMAR) or better, optic nerve cupping < 50%, no glaucoma or retinal diseases, no prior intraocular surgery (excepting non-complicated cataract or refractive surgery performed more than 1 year before), IOP ≤ 20 mmHg, no diabetes, and no diabetic retinopathy as determined by the ophthalmologist or optometrist.

### Color Vision Quantification

Alzheimer’s disease is one of the chronic illnesses that can lead to acquired color vision deficiencies and ultimately, to color blindness (Pache et al., 2003; Cabrera DeBuc et al., 2017). For example, it has been reported that the cone contrast test scores in elderly individuals may be affected by cognitive decline (Simunovic, 2016). Therefore, study subjects were tested for acquired color vision deficiencies considering that the loss in cone function could be caused by neurological, systemic, ocular disorders and trauma to the eye or brain (Simunovic, 2016). A commercially available tablet-based Cone Contrast Test unit (CCT, Provideo CCT Plus System, Innova Systems Inc., Burr Ridge, IL, United States) was used to test the type and severity of color vision deficiency (Rabin et al., 2011). The tablet-based CCT scores, expressed in the range from 0 to 100, were measured on a portable tablet display (10.1^′′^, 1366 × 768 pixels, Windows 8) with a touchscreen interface and the system firmware version 14.2.6. The color vision examination was conducted from the right eye to the left eye in a dark room with the tablet’s display parallel to the individual’s face plane and positioned at near distance (75 cm). The liquid crystal display of the tablet-based CCT was calibrated before the examination. The score results from the left eyes were used when both eyes met the inclusion criteria to lessen potential errors due to inexperience with the CCT test.

**FIGURE 5:**
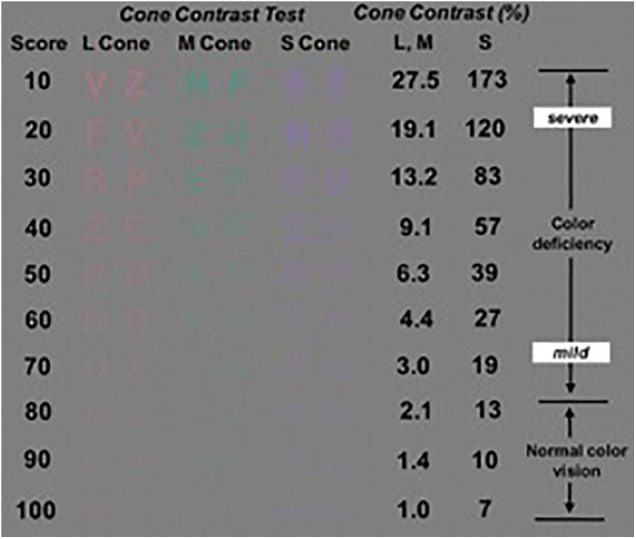
The cone contrast test (CCT) design principles showing the Long, Middle, and Short- CCT scores (i.e., Red, Green and Blue CCT scores, respectively). Image modified from Rabin et al. (2011).

The stimulus displayed by the tablet-based CCT system consisted of a randomized series of colored letters on a gray background which are visible only to Red (R), Green (G), or Blue (B) cones in decreasing steps of cone contrast (Figure 5). During the exam, a single letter is displayed in the center of the screen, and the observer had to use a mouse to select the letter seen from an adjacent 10-letter matching display. Then, based on the observer’ s correct or incorrect responses, color contrast is adjusted up and down using a staircase program, to establish the lowest (least visible) R, G, and B cone contrast that the individual can see. The R, G, and B cone CCT scores are expressed on a scale of 0–100 based on the number of letters identified correctly. As previously reported, Cone contrast test (CCT) scores of 75 or greater were defined as normal (Rabin et al., 2011). Also, the CCT can be used after cataract surgery in elderly patients (Fujikawa et al., 2018).

### Visual Performance Test

The Ceeable Visual Field Analyzer (CVFA) is a cloud-based digital platform used to detect and diagnose retinal disease, and as an aid in monitoring progression of visual disease (Fink, 2004). The CVFA delivers rapid, accurate and low-cost visual testing to patient populations that may not have access to traditional visual testing services. The system is based on the 3D Computer Automated Threshold Amsler Grid (3D-CTAG) test. With one eye covered, the subject is positioned in front of a touch-sensitive computer screen on a head-chin rest and finger-traces the areas of an Amsler grid that are missing from his field of vision. Various degrees of contrast of the Amsler grid are presented by repeating the test at different grayscale levels. The resulting 3D data represent the measured contrast sensitivity across the tested visual field and are stored in a relational MySQL database. The platform includes an automated and integrated artifact removal, analysis, and characterization system, which analyzes 3DCTAG visual field data and objectively identifies and characterizes the occurring visual field defects (scotomas) within according to visual field data transforms and scotoma data transforms. Following each test, a topographical contour map, a 3D depiction of the central hill-of-vision, and the comprehensive visual field and scotoma characterization are automatically generated and displayed onscreen, using the freely available Gnuplot© plotting package.

### Assessment of Cognitive Function

Cognitive function was assessed using the Montreal Cognitive Assessment (MoCA), a widely-used screening test for detecting cognitive impairment (Nasreddine et al., 2005). This brief assessment is a one-page 30-point test administered in about 10–12 min. An experienced examiner performed the test. It focuses on several cognitive domains: short-term memory, visuospatial abilities, executive functions, language abilities, orientation to time and place as well as attention, concentration, and working memory. The MoCA total score range is from 0–30, with lower scores (<26 points) indicating poorer cognitive ability. Patients with a score of ≥ 26 points are generally considered as having normal cognition with an average score of 27.4, compared with 22.1 in people with mild cognitive impairment (MCI) and 16.2 in people with AD (Folstein et al., 1975; Smith et al., 2007). One of the advantages of the MoCA test is that it measures an essential component of dementia (i.e., executive function) that is not measured by the mini-mental state examination (MMSE). It also allows cognitive testing for those who are visually impaired.

### Statistical Analysis

All statistical analyses were performed using IBM SPSS Statistics for Windows, Version 24.0 (IBM Corporation, Armonk, NY, United States). All values are presented as per mean and standard deviation (SD). A p value < 0.05 was considered statistically significant. The Shapiro–Wilk test of normality was used to test the normal distribution of the covariates used in our statistical analysis. The Shapiro–Wilk test did not come out significant for the covariates used in our statistical analysis, hence parametric tests were used. Independent sample *t*-tests were used to compare the means of the variables between the cognitively healthy and the cognitively impaired groups. Pearson product moment correlation was used to find the associations between vascular and functional parameters. Partial correlations were then performed to assess the unique associations between each vascular parameter and the functional parameter while controlling for the other covariates.

## Results

Of the 69 initially recruited participants, 32 had cognitive impairment (46%). We excluded data from individuals that had eyes with poor image quality, AMD, glaucoma, diabetic retinopathy, along with data from a subject with a cardiac pacemaker implanted. Six subjects were pseudophakic but without any ocular history except for cataract surgery. All participants with diabetes mellitus (*n* = 9) and hypertension (*n* = 10) were well controlled and did not exhibit retinopathy signs. After applying all exclusion criteria, a total of 20 subjects with cognitive impairment were included in the final analyses. Table 1 shows the baseline characteristics of these participants. Furthermore, we found that some participants (*n* = 17) with no cognitive impairment had some illnesses (e.g., pre-diabetes, diabetic retinopathy, glaucoma, cataract, AMD, hypothyroidism, controlled HIV, childhood’s eye injury, uncontrolled hypertension, uncontrolled diabetes, and cardiovascular disease) that may share a risk factor with the outcome (i.e., cognitive impairment) under study. Also, although quality of the retinal image was acceptable, an image from one of the cognitively healthy subjects couldn’t be read by the SIVA software for further analysis. Therefore, a total of 19 healthy participants with no cognitive impairment was integrated into a data group after removing the above participants with risk factors and individuals that did not fulfill the age-matching criterion needed for establishing rigorous comparisons with the group of patients with cognitive impairment (Table 1).

**Table 1:** Baseline characteristics of participants with cognitive impairment and cognitively healthy individuals after applying the inclusion/exclusion criteria.

Characteristic	Cognitive impairment (*n* = 20)	Cognitively healthy (*n* = 19)
Mean age, years (*SD*)	81(6)	80(7)
Mean MoCA (*SD*)	17(5)	27(1)
Mean HR [beats per minute, (*SD*)]	72(12)	76(2)
S_p_O_2_	96(2)	98(1)
Male, n (%)	4(20)	3(16)
Ever smoked, n (%)	4(20)	2(10)
Current smoker, n (%)	0(0)	0(0)
Hypertension, n (%)	6(30)	4(20)
Diabetes, n (%)	6(30)	3(16)
Pseudophakic, n (%)	2(10)	4(21)
Dyslipidemia, n (%)	3(15)	0(0)


*MoCA, Montreal Cognitive Assessment.*

Only 6 out of 22 associations were found to be significant. These parameters were the AVR, LDRt, D_0_, D_1_, D_2_ and the IT. The Pearson product moment correlation found significant associations between the vascular parameters and IT (Table 2) but these associations were not significant in the partial correlations when other covariates were controlled for. Partial correlation analysis results are shown in Table 3.

**Table 2:** Statistical significant associations between retinal vascular and functional parameters.

Parameters correlated	Pearson coefficients	*p*-value
AVR vs. IT	0.75	<0.001
LDRt vs. IT	0.48	0.03
D_ o_ vs. IT	0.64	0.002
D_ 1_ vs. IT	0.67	0.001
D_ 2_ vs. IT	0.69	0.001


*AVR, arteriole–venular ratio; IT, implicit time; LDRt, total length-to-diameter ratio (i.e., arteriolar+venular); D_*o*,_ capacity dimension; D_*1*,_ information dimension; D_*2*,_ correlation dimension. For full correlations between all variables, see Supplementary Tables 1–7 in the Supplementary Materials.*

**Table 3:** Associations between retinal vascular and functional parameters after partial correlation analyses.

Parameters correlated	Partial correlation coefficient	*p*-value	Control variables
AVR vs. IT	0.39	0.13	D_o_, D_1_, D_2_, LDRt
LDRt vs. IT	0.15	0.57	D_o_, D_1_, D_2_, AVR
D_o_ vs. IT	0.005	0.98	D_1_, D_2_, AVR, LDRt
D_1_ vs. IT	-0.045	0.87	D_o_, D_2_, AVR, LDRt
D_2_ vs. IT	0.091	0.74	D_o_, D_1_, AVR, LDRt


*For the abbreviations see Table 2.*

**FIGURE 6:**
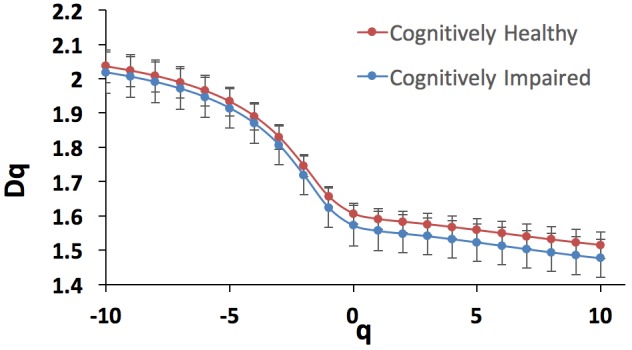
Generalized dimension spectrum D_q_ vs. q for the cognitively healthy individuals (*n* = 19, blue trace) and cognitively impaired (*n* = 20, red trace).

**FIGURE 7:**
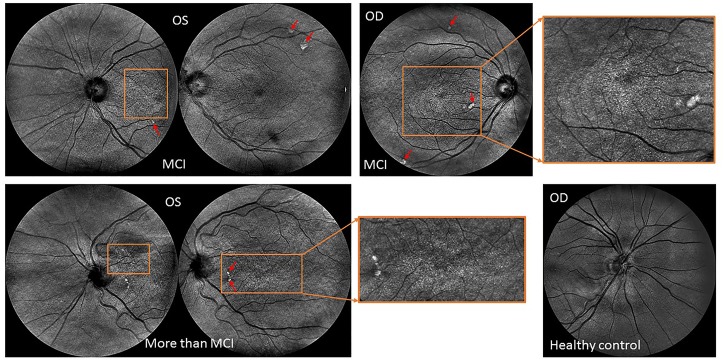
Retinal topographical features observed in individuals with mild cognitive impairment. **(Top row)** Central and nasal infrared light-images obtained from a female subject (79 years old) with MCI showing extramacular features such as drusen-like regions depicted by irregularly shaped bright spots in the periphery of the superior quadrant as well as with pigment dispersion in both eyes. **(Bottom image: Left)** Central and nasal infrared light-images obtained from a female subject (81 years old) with MCI showing tortuous vessels, extramacular features such as drusen-like regions along with pigment dispersion in the left eye. **(Right)** Nasal infrared-light image obtained from a healthy control (71 years old). All images were acquired with the EasyScan Unit (i-Optics Corporation, The Netherlands). The EasyScan camera is a dual color confocal SLO: Infrared (785 nm) and pure green (532 nm). The different colors are related to different penetration depth. ?The red arrows indicate the location of the drusen and white spots observed at extramacular locations. The ROIs enclosed by the orange rectangles indicate the locations where pigment dispersion was observed. The green light-image (see fundus image shown in Figure 1) is reflected at the retinal nerve fiber layer showing the vascular structure up to the 4th bifurcation. The infrared light-image is reaching the choroidal vessel layer.

The fractal analysis was optimized to detect subtle changes in the examined vascular structures (Stosic and Stosic, 2006). Specifically, for all the two sets of images obtained for both the cognitively healthy (*n* = 19) and impaired individuals (*n* = 20), the generalized dimension D_q_ was extracted for different values of q (-10 < q < 10) using the skeletons. Figure 6 shows the generalized dimension spectrum D_q_ vs. independent variable q. As expected, the retinal vascular tree displayed multifractal properties revealed by the descending sigmoid curve Figure 6, giving distinctive FD as the scale was changed. Also, we observed a trend with lower standard deviation (i.e., less oscillation) in cognitively healthy subjects compared to the individuals with cognitively impairment (Figure 7). The calculated mean and standard deviations of generalized dimensions D_0_, D_1_, and D_2_ for both groups are shown in Table 4. Our results also demonstrate that the overall FD is lower that of the DLA (D_q__=__2_ ∼1.71) (Vicsek et al., 1990). The generalized dimensions corresponding to both groups showed a statistically significance difference (Table 4).

**Table 4:** Geometric vascular parameters obtained for patients with cognitive impairment in comparison with the cognitively normal individuals.

Vascular Parameters	Cognitive Impairment Cases *n* = 20 Mean (*SD*)	Cognitively Healthy Cases *n* = 19 Mean (*SD*)	*p*-Value
**Fractal dimension**			
D_0_	1.57 (0.06)	1.61 (0.03)	**0.03^∗^**
D_1_	1.56 (0.06)	1.59 (0.03)	**0.03^∗^**
D_2_	1.55 (0.06)	1.58 (0.03)	**0.02^∗^**
**Caliber (μm)**			
CRAE	65.88 (7.39)	66.73 (6.46)	0.707
CRVE	92.54 (7.15)	92.49 (9.02)	0.984
**Bifurcation**			
BCa	1.65 (0.46)	1.47 (0.35)	0.169
BCv	1.30 (0.48)	1.34 (0.49)	0.812
BCt	1.55 (0.36)	1.50 (0.28)	0.631
AFa	0.75 (0.11)	0.74 (0.16)	0.765
AFv	0.74 (0.22)	0.61 (0.19)	**0.042^∗^**
AFt	0.77 (0.05)	0.71 (0.10)	**0.018^∗^**
**Tortuosity**			
cTORTa (10^-4^)	4.30 (7.04)	4.13 (0.83)	0.485
cTORTv (10^-4^)	4.06 (1.06)	3.82 (0.80)	0.433
cTORTt (10^-4^)	4.17 (0.71)	3.97 (0.64)	0.374
**Ratio measures**			
AVR	0.92 (0.25)	0.86 (0.20)	0.427
LDRa	8.98 (7.46)	7.35 (5.86)	0.456
LDRv	4.52 (6.06)	2.58 (3.77)	0.240
LDRt	9.7 (6.8)	7.13 (4.56)	0.173


*The data reported was measured in the region C (i.e., area between 0.5 and 2.0 disc diameters away from the disc margin, see Figure 1) for all parameters except for the fractal parameters that were calculated in the whole area occupied by the branching pattern (FOV = 45^o^). The *P-*values were calculated by independent sample *t-*test. AVR, arteriole–venular ratio; BCa, arteriolar branching coefficient; BCv, venular branching coefficient; BCt total branching coefficient (i.e., arteriolar+venular); CRAE, central retinal arteriolar equivalent; CRVE, central retinal venular equivalent; LDRa, arteriolar length-to-diameter ratio; LDRv, venular length-to-diameter ratio; LDRt, total length-to-diameter ratio (i.e., arteriolar + venular); cTORTa, curvature arteriolar tortuosity cTORTv, curvature venular tortuosity; cTORTt, total tortuosity (i.e., arteriolar+venular); AFa, asymmetry arteriolar factor; AFv, asymmetry venular factor; Aft, total asymmetry factor (i.e., arteriolar+venular). D_o_: capacity dimension, D_*1*_: information dimension, D_*2*_: correlation dimension. ^∗^Significant (*p* < 0.05).*

We found that the complexity of the branching pattern (FD) of retinal vessels was significantly lower in patients with cognitive impairment in comparison to age-matched controls (see Table 4). As expected the MoCA scores were significantly lower (*p* < 0.001) in the group of cognitive impairment cases compared with the age-matched controls. Although individuals with cognitive impairment showed a trend toward a higher length diameter ratio (LDRa, LDRv, and LDRt), this difference was not statistically significant. We observed that the asymmetry factor was significantly higher in patients with cognitive impairment than in age-matched controls (see Table 4). Also, peripheral drusen-like regions and retinal pigment dispersion were noted in some elderly subjects with MCI (see Figure 7). Geometric vascular parameters and functional measures did not significantly correlate with the MoCA score. For all full-field ERG measurements, smaller amplitudes and larger peak times were observed in the subjects with cognitive impairment (see Table 5). Also, there was a statistical significant (*p* < 0.001) difference in the amplitudes and implicit times between the cognitively healthy group and the one with cognitive deterioration. It is also evident that the implicit time was less variable than the amplitude. Furthermore, the implicit time’s increase that is perceived with the manifestation of pathologic changes of the retina was highly consistent in all patients with cognitive deterioration, and showed practically no overlap between control data and pathologic values: the range of variation seen for control data is between 29.4 and 29.8 ms, while for patients with cognitive impairment it is between 29.6 and 32.8 ms.

**Table 5:** Light-adapted 3.0 flicker ERG (28.3 Hz) measurements recorded from patients with cognitive impairment in comparison with the normative data of the RETeval system.

Light Adapted Test (flicker cone–3.0 cd.s/m^2^, 30 cd/m^2^, 28.3 Hz)	ERG reference data *n* = 244 median [90% CI] Age: [76 – 86]	Cognitive Impairment Cases *n* = 20 median [90% CI] Age: [69 – 90]	Cognitively Healthy Cases *n* = 19 median [90% CI] Age: [60 – 88]	*p*-value
Amplitude(μV)	**2.5% limit** 19.6 [18–22.1] **97.5% limit** 39.4 [35.5–43.1]	12.6 [10.9 – 14.4]	37.4 [36.6 – 38.2]	**<0.001^∗∗^**
Implicit Time(ms)	**2.5% limit** 25.6 [25.1–26.3] **97.5% limit** 29.6 [29.2–29.9]	31.2 [29.6–32.8]	29.6 [29.4 –29.8]	**<0.001^∗∗^**


*Amplitude (μV) and implicit time (ms) are denoted along with the medians and 90% confidence intervals of the 2.5 and 97.5% reference limits. The *P-*values comparing cognitive impairment vs. cognitively healthy cases were calculated by independent sample *t* test. ^∗∗^Significant (*p* < 0.01).*

Visual performance test with the three-dimensional computer-automated version of the threshold Amsler grid test (Ceeable Inc.) demonstrated that this method is subject to variability in the observer’s judgment of the grid threshold for most of the elderly subjects with cognitive impairment. Therefore, because of variability may be exacerbated in these individuals characterized by a pervasive inability to follow detailed task instructions, the visual performance results obtained with the Ceeable platform were not used in the overall analysis. The visual performance test with the computerized Cone Contrast test (CCT, Innova Systems Inc.) revealed functional loss in color vision (see Table 6). There were more patients with more green deficiency than red or blue deficiency. The scores corresponding to both groups showed a statistically significance difference.

**Table 6:** Long, Middle, and Short- CCT scores (i.e., Red, Green, and Blue CCT scores) for the cognitive impairment group.

Rabin CCT scores	Cognitive Impairment Cases *n* = 20 Mean (*SD*)	Cognitively Healthy Cases *n* = 19 Mean (*SD*)	*p*-value
L-CCT (red)	56 (12)	91 (8)	**<0.001^∗∗^**
M-CCT (green)	47 (18)	89 (8)	**<0.001^∗∗^**
S-CCT (blue)	63 (12)	91 (7)	**<0.001^∗∗^**


*CCT scores of 75 or greater are defined as normal (Rabin et al., 2011). The visual performance test with the computerized CCT, revealed functional loss in color vision. The *P-*values were calculated by an independent sample *t-*test. ^∗∗^Significant (*p* < 0.01).*

## Discussion

In this study, multimodal parameters characterizing the structure and function of the retina were compared to evaluate the retinal vascular alterations regarding the retinal function in patients with cognitive impairment. The study was designed to obtain multiple retinal measures, such as structural and functional indicators of the retina. This specific design provided an opportunity to find and study the relationship between various pieces of information, such as the caliber, tortuosity, and network complexity of the retinal microvasculature (arteries and veins) with respect to functional features (e.g., contrast sensitivity, electrical response through ERGs), concomitant with both fractal- vascular and neural analysis.

Retinal vascular attenuation is a well-recognized indicator in patients with cognitive impairment. However, it has not been studied yet in relation to the retinal function, measured by using a low-cost full-field ERG technique. The finding of a significant correlation between the 30 Hz flicker ERG implicit time of the b-wave and AVR, D_0_, D_1_, D_2,_ and LDRt in patients with cognitive decline is intriguing and requires further studies to clarify the underlying pathophysiology and validate its clinical usefulness in predicting the development of cognitive decline using the eye as a surrogate marker. A decrease in amplitude and an increase of the 30 Hz flicker ERG implicit time of the b-wave are usually observed in all retinal pathologies that comprise the photoreceptors when the flicker ERG method has been used to assess photoreceptor function (Meyer et al., 1978; Kondo and Sieving, 2002; Alexander et al., 2006; Verma and Pianta, 2009). Also, previous studies suggest only modest decreases in photopigment optical density with age (Keunen et al., 1987; Elsner et al., 1988; Renner et al., 2004). Therefore, it may be possible that the significant correlations between the ERG parameters and vascular measures could be more related to cognitive decline than aging.

The lack of significant “unique” associations between the vascular parameters and the 30 Hz flicker ERG implicit time may be due to the small sample size in our data as significant trends are seen in the Pearson correlations and these trends may remain significant in the partial correlations in a larger sample size. Secondly, vascular parameters are associated physiologically – for example the vessel branching, artery-vein ratio, and vessel caliber may be related to the complexity of the branching network (FD). Therefore, finding a unique association between these parameters and functional parameters as it was performed with the partial correlations analysis may be statistically robust but in practice, may not follow how the retina is physiologically wired. However, the associations between multiple vascular parameters and functional parameters as shown with the Pearson product moment correlations (Table 2) might describe how the retinal is physiologically wired. Nevertheless, we provide both correlations (Tables 2, 3) to show the statistically significant trend and how this trend changes in the partial correlations.

The CCT scores have been reported to be affected in the elderly due to cognitive decline (Simunovic, 2016). In our study, not only were most patients with cognitive decline found with more green deficiency than red or blue deficiency, but also all CCT scores were severely reduced below the normal decline level (i.e., below a CCT score of 75) associated with aging and reported for the elderly in the eighth and ninth decades of life (i.e., in the 70–79 and 80–89-year age group) (Fujikawa et al., 2018). Although cataract formation may affect the CCT score in phakic eyes of patients in the eighth and ninth decades of life (Fujikawa et al., 2018), the 6 pseudophakic patients in our study were reported to have undergone uneventful cataract surgery with the implantation of a posterior chamber intraocular lens (IOL). Therefore, we believe that these patients with IOL had clear optical media that could not significantly influence our CCT results. Interestingly, it has been reported that individuals with cognitive deterioration due to AD struggle discriminating between green and blue stimuli on the Stroop test which relies on a cognitive measure that requires intact color vision (Cohen et al., 1988; Fisher et al., 1990). These results add to the evidence that extrastriate lesions could result in tritanomalous color deficits (Meadows, 1974; Pearlman et al., 1979), and that the extrastriate cortex is severely disturbed neuropathologically in AD (Lewis et al., 1987). Therefore, pathological changes due to cognitive decline observed in the striate area (IVcß) of the brain that receives color information from the lateral geniculate nucleus, suggest additional basis for deficits in color vision in the brain as described here (Beach and McGeer, 1988).

As in previous studies, we found reduction of vascular branching complexity (FD) in the patients with cognitive decline (Berisha et al., 2007; Frost et al., 2013; Cheung et al., 2014a,b; Ong et al., 2014; Williams et al., 2015). However, compared to these studies, our study used a more robust approach considering the actual multifractal properties of the retinal microvasculature network. Of note, since the findings of AMD and cognitive deterioration due to AD commonalities suggest a degree of overlap (Williams et al., 2014), we assessed all retinal images to identify and rule out retinal pathological features related to AMD. Interestingly, in our patients with cognitive impairment, we observed extramacular drusen in the superior quadrant for some MCI individuals. This trend has been reported previously as to be significantly related with cognitive deterioration due to AD in patients with peripheral drusen (Csincsik et al., 2018). Two earlier studies that may add to this evidence have described the presence of amyloid beta in retinal drusen deposits (Ding et al., 2008; Zhao et al., 2015). Also, abundant amyloid beta pathology has been detected in AD patients in the periphery of the superior quadrant (Koronyo et al., 2017).

Several ERG changes have also been recorded in patients with cognitive deterioration due to AD (Sadun et al., 1987; Armstrong, 1996). Earlier studies have reported that the involvement of the visual cortex may be the cause for dysfunction of the elementary visual sensation that may be involved in the development of visual cognitive deficits and vision-related behavioral symptoms (Strenn et al., 1991; Granholm et al., 2003). The use of the full-field ERG has been suggested to find whether dysfunction of preganglionic elements may also occur in cognitive deterioration due to AD (Parisi et al., 2001). Possible dysfunction of preganglionic elements could explain the increase in P50 implicit time observed in AD patients and this is supported by data obtained in glaucoma or in multiple sclerosis in which the delay of the P50 implicit time could be ascribed to a dysfunction of both ganglionic and preganglionic elements (Katz and Rimmer, 1989; Tobimatsu et al., 1989; Holder, 1997; Porciatti et al., 1997). Moreover, flash ERG was used to demonstrate dysfunction of the retina under photopic and scotopic conditions in patients with dementia with Lewy bodies (Devos et al., 2005). This study outlined that the retinal dysfunction may be related to slight alteration of the photoreceptors and numerous pale inclusions in the outer plexiform layer found at the post-mortem examination, suggesting specific retinopathy (Devos et al., 2005). In our study, we also found a significant reduced a-wave amplitude indicating abnormal photoreceptor function associated to a longer response of the rods under scotopic conditions (Tzekov and Mullan, 2014). The association between the retinal vascular attenuation and the severity of the scotopic full-field alteration have been previously reported in patients with cone degeneration (e.g. retinitis pigmentosa) for which oxidative stress has been suggested to play a potential pathogenic role like in AD (Mecocci et al., 1994; Sandberg et al., 1996; Markesbery, 1997; Wang et al., 2005; Ma et al., 2012). Also, a recent study reported that subretinal injection of amyloid β in C57/BL6 mice yields declined scotopic response (Liu et al., 2015). Interestingly, as in our study, the infrared SLO images revealed drusen-like regions depicted by irregularly shaped bright areas. Moreover, a substantial decrease in mixed rod-cone responses (i.e., decreased a- and b-wave amplitudes) has been noted in mice carrying ApoE-ε 4 allele of apolipoproteine E4 which is the most prevalent genetic risk factor for the late-onset AD that acts in synergy with Aβ (Antes et al., 2013). Consequently, these recent studies on animal models and our preliminary results suggest that evaluation of the bioelectric activity of the retina with ERG may add significant value to the retinal biomarker exploration in cognitive impairment at the early stage. Also, the fact that the 90% confidence intervals of the averages of the cognitively healthy and cognitively impaired groups are not overlapping (Table 5) supports the opportunity to define distinctive domains for the values of the implicit time that can be correlated with the presence and, respectively, the non-existence of cognitive impairment in the individuals analyzed.

A thorough search of the relevant literature yielded no related article reporting retinal vascular and functional abnormalities in cognitive impairment using a multimodal approach that requires an instrumentation cost of less than $45,000. The primary strength of this study is the low-cost multimodal approach implemented to measure combined structural-functional parameters, instead of individual markers. The portability and low-cost of our approach will facilitate to further extend the collection of data in community settings for population health management. Another strength is the multifractal analysis conducted considering that most studies have relied on monofractal analysis; a scheme which has largely attained limited success (Stosic and Stosic, 2006; Azemin et al., 2012).

This study has some important limitations. The exclusion of eyes due to poor image quality because of opacities in the ocular media (e.g., cataracts and floaters in some elderly subjects), and the presence of confounding factors limited the sample size. Also, we found some challenges in achieving both structural and functional data with high quality, mainly in elderly patients with cognitive impairment who are easily fatigued. These challenges can be more complex if the study subject has poor vision. Also, we did not examine the relationship between the vast number of parameters in the SIVA platform and the functional parameters because of small sample size. Besides the complexity of the branching pattern of retinal vessels, AVR, asymmetry ratios, LDR, branching coefficients, tortuosity and vessel caliber, it is possible that other geometric vascular parameters (e.g., other bifurcation parameters, branching coefficient angle, etc.) are associated with physiological changes in the retina due to cognitive decline and that the functional parameters are sensitive to these changes. Another limitation could be that the cognitive function of the study patients was only assessed by the MoCA test. Therefore, differential diagnosis of cognitive impairment was not possible, and detailed evaluations of cognitive functions including pathological examinations and neuroimaging of the brain will be needed to confirm the existence of cognitive deterioration due to AD. However, although AD is the most common, progressive cause of dementia in the elderly, our study recruited subjects independently of their cognitive impairment’s causation which helped us to assess the collection of data in community settings for population health management. It has been reported that amyloid deposition can be identified among cognitively normal elderly persons during life and the prevalence of asymptomatic amyloid deposition may be like that of symptomatic amyloid deposition. An early study has shown that in a group of participants without clinically significant impairment, amyloid deposition was not associated with worse cognitive function, suggesting that an elderly person with a significant amyloid burden can remain cognitively normal (Aizenstein et al., 2008). Pagani et al. (2016) has also shown that there is no deterministic relation between cognitive impairment and AD severity. Nevertheless, a longitudinal follow-up of study subjects would be required to support the potential of amyloid imaging to identify preclinical Alzheimer disease or alternatively, to show that amyloid deposition is not sufficient to cause Alzheimer disease within some specified period. Therefore, our multivariate and multimodal approach using an agnostic-cognitive impairment assessment could be a starting point for expanding the methodology in community settings to assess the eye-brain conditions of individuals under the risk of cognitive deterioration.

Although significant correlations between the functional and vascular parameters did not survive after the partial correlations analyses, that does not imply the statistical trends found in this study are not revealing of alteration and disease of the neuro-vascular component in general. Also, the cross-sectional setting of our study couldn’t facilitate the investigation of temporal and causal relationships between the retinal functional and structural features with cognitive impairment. However, due to the strict exclusion criteria used in our study, we only analyzed very good quality data that makes our results more robust. Despite the above limitations, the retinal vascular attenuation and reduced complexity of the vascular branching network is comparable to those observed in earlier studies (Berisha et al., 2007; Frost et al., 2013; Cheung et al., 2014a,b). We continue collecting data under this study and expect to explore the multiple relationships and statistical trends further with larger sample size.

## Conclusion

The difficulty in detecting cognitive impairment in its early stages poses a limitation on the onset of cognitive decline diagnosis. Unfortunately, there is no successful treatment once early cognitive impairment or dementia becomes clinically apparent (Hampel et al., 2018). This study illustrates that there are multimodal retinal markers that may be sensitive to cognitive impairment decline, and adds to the evidence that there is a statistical trend pointing to the correlation between retinal neuronal dysfunction and microvasculature changes. This trend suggests that retinal geometric vascular and functional parameters might be associated with physiological changes in the retina due to cognitive decline. We suspect our analysis of combined structural-functional parameters, instead of individual biomarkers, may serve as a useful clinical marker of cognitive decline that could also provide increased sensitivity and specificity for the differential diagnosis of cognitive impairment. However, because of our study sample was small, the full extent of clinical applicability of our approach is provocative and still to be determined. This study also adds support to the use of a multimodal diagnostic biomarker approach of cognitive impairment based on the retinal structure-function relationship which also has the advantage of a low-cost implementation in community settings to detect cognitive decline-specific pathology in the retina, which could enable the early diagnosis and monitoring of disease progression. Provided a clinical correlation between the eye and brain measures can be confirmed, screening of eyes in people being considered at risk of cognitive impairment could help in the development of an alternative low-cost approach for early diagnosis as well as potentially serve to monitor the effectiveness of emerging therapies.

## Author Contributions

DCD conceived and designed the study. DCD, GS, EA, MK, SO, and CMS performed the study. DCD, GS, EA, and SO analyzed the data. DCD and GS contributed to reagents, materials, and analysis tools. DCD, GS, and EA contributed to the writing of the manuscript.

## Conflict of Interest Statement

The authors declare that the research was conducted in the absence of any commercial or financial relationships that could be construed as a potential conflict of interest.

## References

[B1] Aizenstein H. J. Nebes R. D. Saxton J. A. Price J. C. Mathis C. A. Tsopelas N. D. (2008). Frequent amyloid deposition without significant cognitive impairment among the elderly. *Arch. Neurol.* 65 1509–1517. 10.1001/archneur.65.11.1509 19001171 PMC2636844

[B2] Alexander K. R. Rajagopalan A. S. Raghuram A. Fishman G. A. (2006). Activation phase of cone phototransduction and the flicker electroretinogram in retinitis pigmentosa. *Vision Res.* 46 2773–2785. 10.1016/j.visres.2006.01.007 16494917

[B3] Antes R. Ezra-Elia R. Weinberger D. Solomon A. Ofri R. Michaelson D. M. (2013). ApoE4 induces synaptic and ERG impairments in the retina of young targeted replacement apoE4 mice. *PLoS One* 8:e64949. 10.1371/journal.pone.0064949 23741431 PMC3669199

[B4] Armstrong R. A. (1996). Visual field defects in Alzheimer’s disease patients may reflect differential pathology in the primary visual cortex. *Optom. Vis. Sci.* 73 677–682. 10.1097/00006324-199611000-00001 8950748

[B5] Azemin M. Z. Kumar D. K. Wong T. Y. Wang J. J. Mitchell P. Kawasaki R. (2012). Age-related rarefaction in the fractal dimension of retinal vessel. *Neurobiol. Aging.* 33 194.e1–194.e4. 10.1016/j.neurobiolaging.2010.04.010 20472327

[B6] Beach T. G. McGeer E. G. (1988). Lamina-specific arrangement of astrocytic gliosis and senile plaques in Alzheimer’s disease visual cortex. *Brain Res.* 463 357–361. 10.1016/0006-8993(88)90410-6 3196922

[B7] Berisha F. Feke G. T. Trempe C. L. Mcmeel J. W. Schepens C. L. (2007). Retinal abnormalities in early Alzheimer’s disease. *Invest. Ophthalmol. Vis. Sci.* 48 2285–2289. 10.1167/iovs.06-1029 17460292

[B8] Braak H. Braak E. (1997). Frequency of stages of Alzheimer-related lesions in different age categories. *Neurobiol. Aging.* 18 351–357. 10.1016/S0197-4580(97)00056-0 9330961

[B9] Cabrera DeBuc D. Somfai G. M. Koller A. (2017). Retinal microvascular network alterations: potential biomarkers of cerebrovascular and neural diseases. *Am. J. Physiol. Heart. Circ. Physiol.* 312 H201–H212. 10.1152/ajpheart.00201.2016 27923786 PMC5336575

[B10] Cheung C. Y. Ong S. Ikram M. K. Ong Y. T. Chen C. P. Venketasubramanian N. (2014a). Retinal vascular fractal dimension is associated with cognitive dysfunction. *J. Stroke Cerebrovasc. Dis.* 23 43–50. 10.1016/j.jstrokecerebrovasdis.2012.09.002 23099042

[B11] Cheung C. Y. Ong Y. T. Ikram M. K. Ong S. Y. Li X. Hilal S. (2014b). Microvascular network alterations in the retina of patients with Alzheimer’s disease. *Alzheimers Dement.* 10 135–142. 10.1016/j.jalz.2013.06.009 24439169

[B12] Cheung C. Y. Tay W. T. Mitchell P. Wang J. J. Hsu W. Lee M. L. (2011). Quantitative and qualitative retinal microvascular characteristics and blood pressure. *J. Hypertens.* 29 1380–1391. 10.1097/HJH.0b013e328347266c 21558958

[B13] Cohen J. Cronin-Golomb A. Growdon J. H. Corkin S. (1988). Colour vision deficits in Alzheimer’s disease. *Soc. Neurosci. Abstr.* 14:219.

[B14] Cosatto V. F. Liew G. Rochtchina E. Wainwright A. Zhang Y. Hsu W. (2010). Retinal vascular fractal dimension measurement and its influence from imaging variation: results of two segmentation methods. *Curr. Eye Res.* 35 850–856. 10.3109/02713683.2010.490628 20795868

[B15] Csincsik L. Macgillivray T. J. Flynn E. Pellegrini E. Papanastasiou G. Barzegar-Befroei N. (2018). Peripheral retinal imaging biomarkers for Alzheimer’s disease: a pilot study. *Ophthalmic Res.* 59 182–192. 10.1159/000487053 29621759 PMC5985743

[B16] Devos D. Tir M. Maurage C. A. Waucquier N. Defebvre L. Defoort-Dhellemmes S. (2005). ERG and anatomical abnormalities suggesting retinopathy in dementia with Lewy bodies. *Neurology* 65 1107–1110. 10.1212/01.wnl.0000178896.44905.33 16217068

[B17] Ding J. D. Lin J. Mace B. E. Herrmann R. Sullivan P. Bowes Rickman C. (2008). Targeting age-related macular degeneration with Alzheimer’s disease based immunotherapies: anti-amyloid-beta antibody attenuates pathologies in an age-related macular degeneration mouse model. *Vision Res.* 48 339–345. 10.1016/j.visres.2007.07.025 17888483 PMC2323206

[B18] Elias M. F. Beiser A. Wolf P. A. Au R. White R. F. D’agostino R. B. (2000). The preclinical phase of alzheimer disease: a 22-year prospective study of the Framingham Cohort. *Arch. Neurol.* 57 808–813. 10.1001/archneur.57.6.808 10867777

[B19] Elsner A. E. Berk L. Burns S. A. Rosenberg P. R. (1988). Aging and human cone photopigments. *J. Opt. Soc. Am. A.* 5 2106–2112. 10.1364/JOSAA.5.002106 3230479

[B20] Family F. Masters B. R. Platt D. E. (1989). Fractal pattern formation in human retinal vessels. *Phys. D Nonlinear Phenom.* 38 98–103. 10.1016/0167-2789(89)90178-4

[B21] Fink W. (2004). Neural attractor network for application in visual field data classification. *Phys. Med. Biol.* 49 2799–2809. 10.1088/0031-9155/49/13/003 15285248

[B22] Fisher L. M. Freed D. M. Corkin S. (1990). Stroop color-word test performance in patients with Alzheimer’s disease. *J. Clin. Exp. Neuropsychol.* 12 745–758. 10.1080/01688639008401016 2258434

[B23] Folstein M. F. Folstein S. E. Mchugh P. R. (1975). “Mini-mental state”. A practical method for grading the cognitive state of patients for the clinician. *J. Psychiatr. Res.* 12 189–198. 10.1016/0022-3956(75)90026-6 1202204

[B24] Frost S. Kanagasingam Y. Sohrabi H. Vignarajan J. Bourgeat P. Salvado O. (2013). Retinal vascular biomarkers for early detection and monitoring of Alzheimer’s disease. *Transl. Psychiatry* 3:e233. 10.1038/tp.2012.150 23443359 PMC3591002

[B25] Fujikawa M. Muraki S. Niwa Y. Ohji M. (2018). Evaluation of clinical validity of the Rabin cone contrast test in normal phakic or pseudophakic eyes and severely dichromatic eyes. *Acta Ophthalmol.* 96 e164–e167. 10.1111/aos.13495 28556475 PMC5836892

[B26] Gould D. J. Vadakkan T. J. Poché R. A. Dickinson M. E. (2011). Multifractal and lacunarity analysis of microvascular morphology and remodeling. *Microcirculation* 18 136–151. 10.1111/j.1549-8719.2010.00075.x 21166933 PMC3049800

[B27] Granholm E. Morris S. Galasko D. Shults C. Rogers E. Vukov B. (2003). Tropicamide effects on pupil size and pupillary light reflexes in Alzheimer’s and Parkinson’s disease. *Int. J. Psychophysiol.* 47 95–115. 10.1016/S0167-8760(02)00122-8 12568941

[B28] Hampel H. Toschi N. Babiloni C. Baldacci F. Black K. L. Bokde A. L. W. (2018). Revolution of Alzheimer precision neurology. Passageway of systems biology and neurophysiology. *J. Alzheimers Dis.* 64 S47–S105. 10.3233/JAD-179932 29562524 PMC6008221

[B29] Hart W. E. Goldbaum M. Cote B. Kube P. Nelson M. R. (1999). Measurement and classification of retinal vascular tortuosity. *Int. J. Med. Inform.* 53 239–252. 10.1016/S1386-5056(98)00163-4 10193892

[B30] Holder G. E. (1997). The pattern electroretinogram in anterior visual pathway dysfunction and its relationship to the pattern visual evoked potential: a personal clinical review of 743 eyes. *Eye (Lond.)* 11 924–934. 10.1038/eye.1997.231 9537157

[B31] Holder G. E. Brigell M. G. Hawlina M. Meigen T. Vaegan Bach M. International Society for Clinical Electrophysiology of Vision (2007). ISCEV standard for clinical pattern electroretinography–2007 update. *Doc. Ophthalmol.* 114 111–116. 10.1007/s10633-007-9053-1 17435967 PMC1896293

[B32] Hood D. C. Bach M. Brigell M. Keating D. Kondo M. Lyons J. S. (2008). ISCEV guidelines for clinical multifocal electroretinography (2007 edition). *Doc. Ophthalmol.* 116 1–11. 10.1007/s10633-007-9089-2 17972125 PMC2235911

[B33] Karperien A. (1993). FracLac for ImageJ— FracLac Advanced User’s Manual. Bethesda, MD: National Institutes of Health Available : http://rsb.info.nih.gov/ij/plugins/fraclac/fraclac-manual.pdf (accessed August 20, 2018).

[B34] Kato K. Kondo M. Sugimoto M. Ikesugi K. Matsubara H. (2015). Effect of pupil size on flicker ERGs recorded with RETeval system: new mydriasis-free full-field ERG system. *Invest. Ophthalmol. Vis. Sci.* 56 3684–3690. 10.1167/iovs.14-16349 26047169

[B35] Katz B. Rimmer S. (1989). Ophthalmologic manifestations of Alzheimer’s disease. *Surv. Ophthalmol.* 34 31–43. 10.1016/0039-6257(89)90127-6 2678551

[B36] Kawas C. H. Corrada M. M. Brookmeyer R. Morrison A. Resnick S. M. Zonderman A. B. (2003). Visual memory predicts Alzheimer’s disease more than a decade before diagnosis. *Neurology* 60 1089–1093. 10.1212/01.WNL.0000055813.36504.BF 12682311

[B37] Keunen J. E. Van Norren D. Van Meel G. J. (1987). Density of foveal cone pigments at older age. *Invest. Ophthalmol. Vis. Sci.* 28 985–991. 3583637

[B38] Kondo M. Sieving P. A. (2002). Post-photoreceptoral activity dominates primate photopic 32-Hz ERG for sine-, square-, and pulsed stimuli. *Invest. Ophthalmol. Vis. Sci.* 43 2500–2507. 12091456

[B39] Koronyo Y. Biggs D. Barron E. Boyer D. S. Pearlman J. A. Au W. J. (2017). Retinal amyloid pathology and proof-of-concept imaging trial in Alzheimer’s disease. *JCI Insight* 2:93621. 10.1172/jci.insight.93621 28814675 PMC5621887

[B40] Koronyo Y. Salumbides B. C. Black K. L. Koronyo-Hamaoui M. (2012). Alzheimer’s disease in the retina: imaging retinal abeta plaques for early diagnosis and therapy assessment. *Neurodegener. Dis.* 10 285–293. 10.1159/000335154 22343730

[B41] Koronyo-Hamaoui M. Koronyo Y. Ljubimov A. V. Miller C. A. Ko M. K. Black K. L. (2011). Identification of amyloid plaques in retinas from Alzheimer’s patients and noninvasive in vivo optical imaging of retinal plaques in a mouse model. *Neuroimage* 54 S204–S217. 10.1016/j.neuroimage.2010.06.020 20550967 PMC2991559

[B42] Kyriacos S. Nekka F. Vicco P. Cartilier L. (1997). “The retinal vasculature: towards an understanding of the formation process,” in *Fractals in Engineering*, eds Vehel L. J. Lutton E. Tricot C. (London: Springer), 383–397.

[B43] La Rue A. Jarvik L. F. (1987). Cognitive function and prediction of dementia in old age. *Int. J. Aging Hum. Dev.* 25 79–89. 10.2190/DV3R-PBJQ-E0FT-7W2B 3436685

[B44] Lavoie J. Illiano P. Sotnikova T. D. Gainetdinov R. R. Beaulieu J. M. Hebert M. (2014). The electroretinogram as a biomarker of central dopamine and serotonin: potential relevance to psychiatric disorders. *Biol. Psychiatry* 75 479–486. 10.1016/j.biopsych.2012 23305992

[B45] Lewis D. A. Campbell M. J. Terry R. D. Morrison J. H. (1987). Laminar and regional distributions of neurofibrillary tangles and neuritic plaques in Alzheimer’s disease: a quantitative study of visual and auditory cortices. *J. Neurosci.* 7 1799–1808. 10.1523/JNEUROSCI.07-06-01799.1987 2439665 PMC6568896

[B46] Liebovitch L. S. Toth T. (1989). A fast algorithm to determine fractal dimensions by box counting. *Phys. Lett. A* 141 386–390. 10.1016/0375-9601(89)90854-2

[B47] Liew G. Wang J. J. Cheung N. Zhang Y. P. Hsu W. Lee M. L. (2008). The retinal vasculature as a fractal: methodology, reliability, and relationship to blood pressure. *Ophthalmology* 115 1951–1956. 10.1016/j.ophtha.2008.05.029 18692247

[B48] Linn R. T. Wolf P. A. Bachman D. L. Knoefel J. E. Cobb J. L. Belanger A. J. (1995). The ‘preclinical phase’ of probable Alzheimer’s disease. A 13-year prospective study of the Framingham cohort. *Arch. Neurol.* 52 485–490. 10.1001/archneur.1995.00540290075020 7733843

[B49] Liu C. Cao L. Yang S. Xu L. Liu P. Wang F. (2015). Subretinal injection of amyloid-beta peptide accelerates RPE cell senescence and retinal degeneration. *Int. J. Mol. Med.* 35 169–176. 10.3892/ijmm.2014.1993 25385658

[B50] London A. Benhar I. Schwar M. (2012). The retina as a window to the brain-from eye research to CNS disorders. *Nat. Rev. Neurol.* 9 44–53. 10.1038/nrneurol.2012.227 23165340

[B51] Ma Y. Kawasaki R. Dobson L. P. Ruddle J. B. Kearns L. S. Wong T. Y. (2012). Quantitative analysis of retinal vessel attenuation in eyes with retinitis pigmentosa. *Invest. Ophthalmol. Vis. Sci.* 53 4306–4314. 10.1167/iovs.11-8596 22661482

[B52] Macgillivray T. J. Patton N. Doubal F. N. Graham C. Wardlaw J. M. (2007). Fractal analysis of the retinal vascular network in fundus images. *Conf. Proc. IEEE. Eng. Med. Biol. Soc.* 2007 6456–6459. 10.1109/IEMBS.2007.4353837 18003503

[B53] Mainster M. A. (1990). The fratal properties of retinal vessels: embryological and clinical implications. *Eye (Lond.)* 4 235–241. 10.1038/eye.1990.33 2323476

[B54] Mandelbrot B. B. (1982). *The Fractal Geometry of Nature.* New York, NY: W.H. Freeman.

[B55] Markesbery W. R. (1997). Oxidative stress hypothesis in Alzheimer’s disease. *Free Radic. Biol. Med.* 23 134–147. 10.1016/S0891-5849(96)00629-6 9165306

[B56] Marmor M. F. Holder G. E. Seeliger M. W. Yamamoto S. International Society for Clinical Electrophysiology Of Vision (2004). Standard for clinical electroretinography (2004 update). *Doc. Ophthalmol.* 108 107–114. 10.1023/B:DOOP.0000036793.44912.45 15455793

[B57] McCulloch D. L. Marmor M. F. Brigell M. G. Hamilton R. Holder G. E. Tzekov R. (2015). ISCEV standard for full-field clinical electroretinography (2015 update). *Doc. Ophthalmol.* 130 1–12. 10.1007/s10633-014-9473-7 25502644

[B58] Meadows J. C. (1974). Disturbed perception of colours associated with localized cerebral lesions. *Brain* 97 615–632. 10.1093/brain/97.1.615 4547992

[B59] Mecocci P. Macgarvey U. Beal M. F. (1994). Oxidative damage to mitochondrial DNA is increased in Alzheimer’s disease. *Ann. Neurol.* 36 747–751. 10.1002/ana.410360510 7979220

[B60] Meyer J. J. Korol S. Gramoni R. Tupling R. (1978). Psychophysical flicker thresholds and ERG flicker responses in congenital and acquired vision deficiencies. *Mod. Probl. Ophthalmol.* 19 33–49. 310055

[B61] Moschos M. M. Markopoulos I. Chatziralli I. Rouvas A. Papageorgiou S. G. Ladas I. (2012). Structural and functional impairment of the retina and optic nerve in Alzheimer’s disease. *Curr. Alzheimer Res.* 9 782–788. 10.2174/156720512802455340 22698074

[B62] Nasreddine Z. S. Phillips N. A. Bedirian V. Charbonneau S. Whitehead V. Collin I. (2005). The montreal cognitive assessment, MoCA: a brief screening tool for mild cognitive impairment. *J. Am. Geriatr. Soc.* 53 695–699. 10.1111/j.1532-5415.2005.53221.x 15817019

[B63] Ong Y. T. Hilal S. Cheung C. Y. Xu X. Chen C. Venketasubramanian N. (2014). Retinal vascular fractals and cognitive impairment. *Dement. Geriatr. Cogn. Dis. Extra* 4 305–313. 10.1159/000363286 25298774 PMC4176466

[B64] Pache M. Smeets C. H. Gasio P. F. Savaskan E. Flammer J. Wirz-Justice A. (2003). Colour vision deficiencies in Alzheimer’s disease. *Age Ageing* 32 422–426. 10.1093/ageing/32.4.422 12851187

[B65] Pagani M. Giuliani A. Oberg J. Chincarini A. Morbelli S. Brugnolo A. (2016). Predicting the transition from normal aging to Alzheimer’s disease: a statistical mechanistic evaluation of FDG-PET data. *Neuroimage* 141 282–290. 10.1016/j.neuroimage.2016.07.043 27453158

[B66] Parisi V. Restuccia R. Fattapposta F. Mina C. Bucci M. G. Pierelli F. (2001). Morphological and functional retinal impairment in Alzheimer’s disease patients. *Clin. Neurophysiol.* 112 1860–1867. 10.1016/S1388-2457(01)00620-4 11595144

[B67] Pearlman A. L. Birch J. Meadows J. C. (1979). Cerebral color blindness: an acquired defect in hue discrimination. *Ann. Neurol.* 5 253–261. 10.1002/ana.410050307 312619

[B68] Prince M. Wimo A. Guerchet M. Ali G. C. Wu Y. T. Prina M. (2015). *World Alzheimer Report 2015. The Global Impact of Dementia.* London: Alzheimer’s Disease International (ADI).

[B69] Porciatti V. Di Bartolo E. Nardi M. Fiorentini A. (1997). Responses to chromatic and luminance contrast in glaucoma: a psychophysical and electrophysiological study. *Vision. Res.* 37 1975–1987. 10.1016/S0042-6989(97)00018-7 9274782

[B70] Rabin J. Gooch J. Ivan D. (2011). Rapid quantification of color vision: the cone contrast test. *Invest. Ophthalmol. Vis. Sci.* 52 816–820. 10.1167/iovs.10-6283 21051721

[B71] Renner A. B. Knau H. Neitz M. Neitz J. Werner J. S. (2004). Photopigment optical density of the human foveola and a paradoxical senescent increase outside the fovea. *Vis. Neurosci.* 21 827–834. 10.1017/S0952523804216030 15733338 PMC2603297

[B72] Sadun A. A. Borchert M. Devita E. Hinton D. R. Bassi C. J. (1987). Assessment of visual impairment in patients with Alzheimer’s disease. *Am. J. Ophthalmol.* 104 113–120. 10.1016/0002-9394(87)90001-8 3618708

[B73] Sandberg M. A. Weigel-Difranco C. Rosner B. Berson E. L. (1996). The relationship between visual field size and electroretinogram amplitude in retinitis pigmentosa. *Invest. Ophthalmol. Vis. Sci.* 37 1693–1698. 8675413

[B74] Simunovic M. P. (2016). Acquired color vision deficiency. *Surv. Ophthalmol.* 61 132–155. 10.1016/j.survophthal.2015.11.004 26656928

[B75] Smith T. Gildeh N. Holmes C. (2007). The montreal cognitive assessment: validity and utility in a memory clinic setting. *Can. J. Psychiatry* 52 329–332. 10.1177/070674370705200508 17542384

[B76] Snowdon D. A. Kemper S. J. Mortimer J. A. Greiner L. H. Wekstein D. R. Markesbery W. R. (1996). Linguistic ability in early life and cognitive function and Alzheimer’s disease in late life. Findings from the Nun Study. *JAMA* 275 528–532. 10.1001/jama.1996.03530310034029 8606473

[B77] Stosic T. Stosic B. D. (2006). Multifractal analysis of human retinal vessels. *IEEE. Trans. Med. Imaging* 25 1101–1107. 10.1109/TMI.2006.879316 16895002

[B78] Strenn K. Dal-Bianco P. Weghaupt H. Koch G. Vass C. Gottlob I. (1991). Pattern electroretinogram and luminance electroretinogram in Alzheimer’s disease. *J. Neural. Transm. Suppl.* 33 73–80. 10.1007/978-3-7091-9135-4_12 1753255

[B79] Sun C. Wang J. J. Mackey D. A. Wong T. Y. (2009). Retinal vascular caliber: systemic, environmental, and genetic associations. *Surv. Ophthalmol.* 54 74–95. 10.1016/j.survophthal.2008.10.003 19171211

[B80] Ţălu Ş. (2013a). Characterization of retinal vessel networks in human retinal imagery using quantitative descriptors. *Hum. Vet. Med. Bioflux* 5 52–57.

[B81] Ţălu Ş. (2013b). Multifractal geometry in analysis and processing of digital retinal photographs for early diagnosis of human diabetic macular edema. *Curr. Eye Res.* 38 781–792. 10.3109/02713683.2013.779722 23537336

[B82] Thomas G. N. Ong S. Y. Tham Y. C. Hsu W. Lee M. L. Lau Q. P. (2014). Measurement of macular fractal dimension using a computer-assisted program. *Invest. Ophthalmol. Vis. Sci.* 55 2237–2243. 10.1167/iovs.13-13315 24526443

[B83] Tobimatsu S. Celesia G. G. Cone S. Gujrati M. (1989). Electroretinograms to checkerboard pattern reversal in cats: physiological characteristics and effect of retrograde degeneration of ganglion cells. *Electroencephalogr. Clin. Neurophysiol.* 73 341–352. 10.1016/0013-4694(89)90112-0 2477220

[B84] Tzekov R. Mullan M. (2014). Vision function abnormalities in Alzheimer disease. *Surv. Ophthalmol.* 59 414–433. 10.1016/j.survophthal.2013.10.002 24309127

[B85] Vehel J. L Legrand P. (2003). “Bayesian multifractal signal denoising. IEEE 6, VI-177,” in *IEEE International Conference on Acoustics, Speech, and Signal Processing* (Accession No. 7816409), Brighton 10.1109/ICASSP.2003.1201647

[B86] Verma R. Pianta M. J. (2009). The contribution of human cone photoreceptors to the photopic flicker electroretinogram. *J. Vis.* 9 9 1–12. 10.1167/9.3.9 19757948

[B87] Vicsek T. Family F. Meakin P. (1990). Multifractal geometry of diffusion-limited aggregates. *Europhys. Lett.* 12 217–222. 10.1209/0295-5075/12/3/005

[B88] Wang J. Xiong S. Xie C. Markesbery W. R. Lovell M. A. (2005). Increased oxidative damage in nuclear and mitochondrial DNA in Alzheimer’s disease. *J. Neurochem.* 93 953–962. 10.1111/j.1471-4159.2005.03053.x 15857398

[B89] Webb R. H. Hughes G. W. Delori F. C. (1987). Confocal scanning laser ophthalmoscope. *Appl. Opt.* 26 1492–1499. 10.1364/AO.26.001492 20454349

[B90] Williams M. A. Mcgowan A. J. Cardwell C. R. Cheung C. Y. Craig D. Passmore P. (2015). Retinal microvascular network attenuation in Alzheimer’s disease. *Alzheimers Dement. (Amst).* 1 229–235. 10.1016/j.dadm.2015.04.001 26634224 PMC4629099

[B91] Williams M. A. Silvestri V. Craig D. Passmore A. P. Silvestri G. (2014). The prevalence of age-related macular degeneration in Alzheimer’s disease. *J. Alzheimers Dis.* 42 909–914. 10.3233/JAD-140243 25024309

[B92] Witten T. A. Sander L. M. (1981). Diffusion-limited aggregation: a kinetic critical phenomenon. *Phys. Rev. Lett.* 47 1400–1403. 10.1103/PhysRevLett.47.1400

[B93] Zamir M. Medeiros J. A. Cunningham T. K. (1979). Arterial bifurcations in the human retina. *J. Gen. Physiol.* 74 537–548. 10.1085/jgp.74.4.537 512630 PMC2228563

[B94] Zhao Y. Bhattacharjee S. Jones B. M. Hill J. M. Clement C. Sambamurti K. (2015). Beta-amyloid precursor protein (betaAPP) processing in Alzheimer’s disease (AD) and age-related macular degeneration (AMD). *Mol. Neurobiol.* 52 533–544. 10.1007/s12035-014-8886-3 25204496 PMC4362880

