# Supplementary material for: Investigating Multimodal Diagnostic Eye Biomarkers of Cognitive Impairment by Measuring Vascular and Neurogenic Changes in the Retina
Source: Front Physiol. 2018 Dec 6;9:1721. doi: 10.3389/fphys.2018.01721 (PMC6291749; doi:10.3389/fphys.2018.01721)
Supplement: Supplementary file 1 [file Data_Sheet_1.PDF]

**Supplementary Information: Double log plots of the remaining cognitively impaired participants not shown in the manuscript. The subsequent pages contain a plot of the log of the number of boxes containing meaningful pixels vs. the log of the box sizes with their corresponding slope and $R^2$ values. The slope represents $D_0$ (counting box fractal dimension) and the $R^2$ values shows the quality of the regression plots. Each page contains plots for two subjects.**

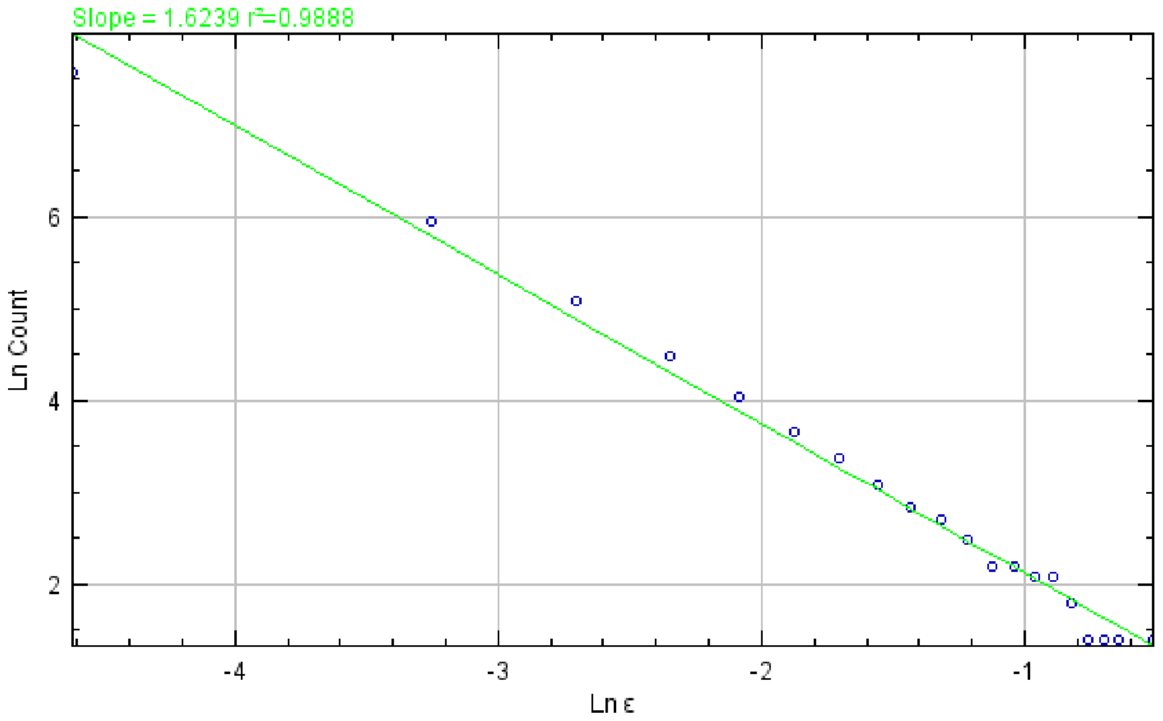
Slope = 1.6239 r²=0.9888
Ln Count
Ln ε
-4
-3
-2
-1
6
4
2

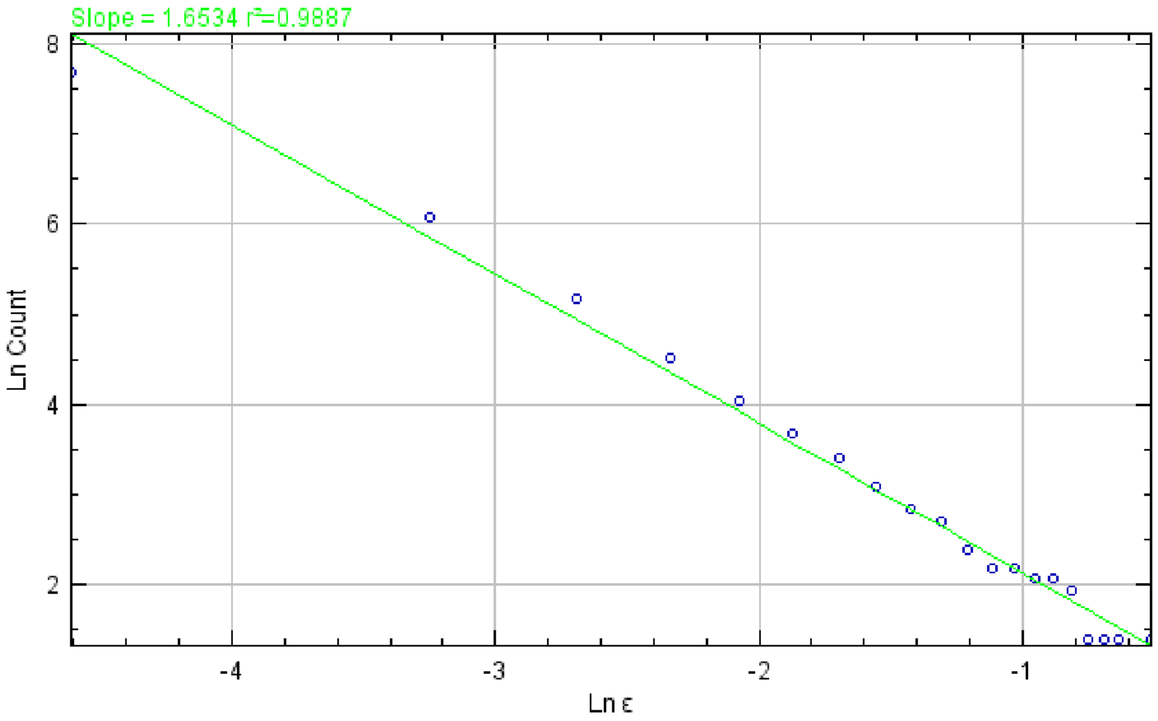
Slope = 1.6534 r²=0.9887
Ln Count
Ln ε
-4
-3
-2
-1
8
6
4
2

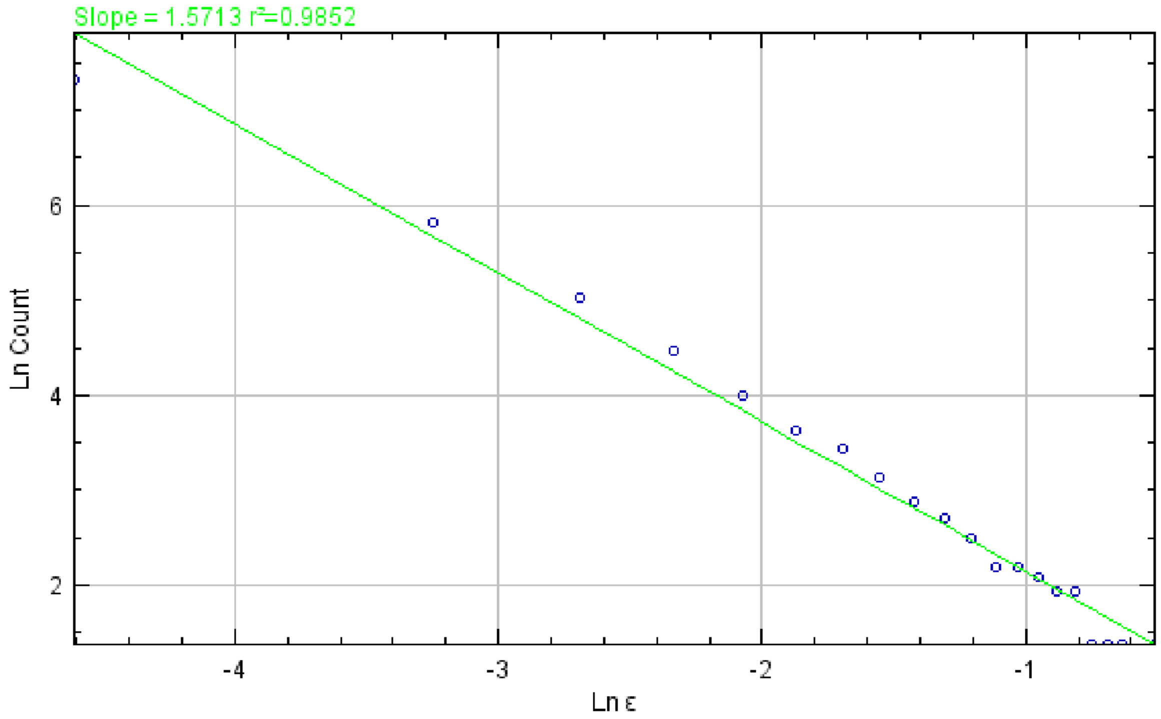
Slope = 1.5713 r²=0.9852
Ln Count
Ln ε
6
4
2
-4
-3
-2
-1

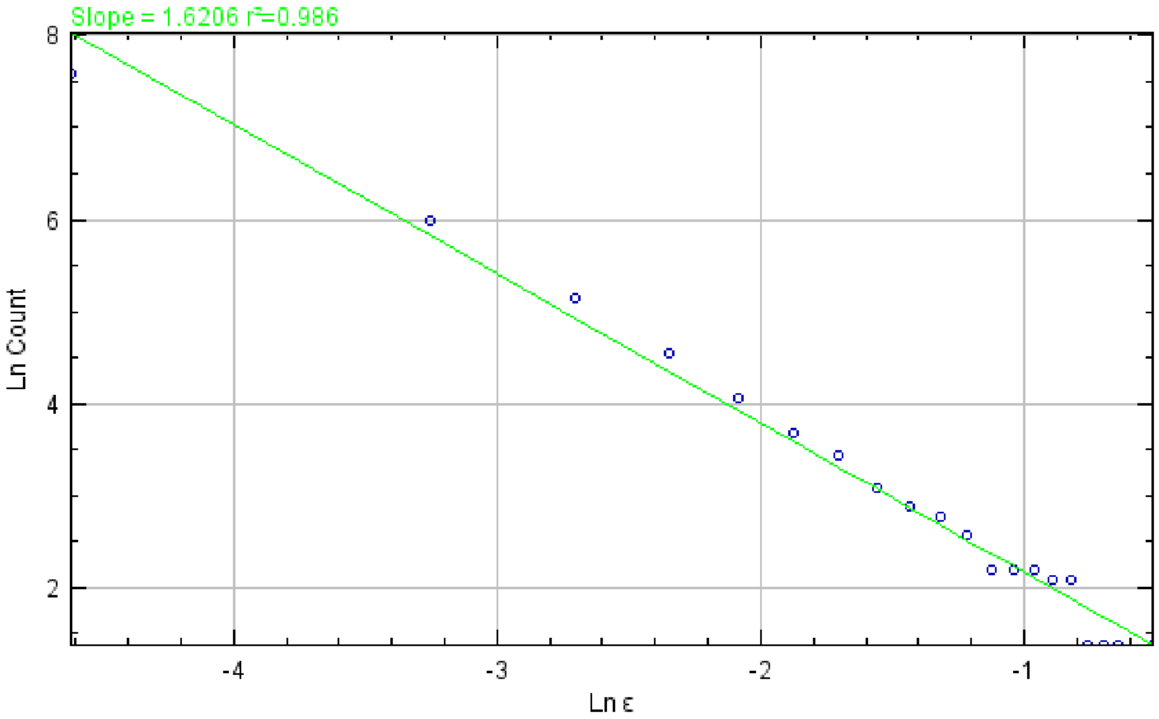
Slope = 1.6206 r²=0.986
Ln Count
Ln ε
8
6
4
2
-4
-3
-2
-1

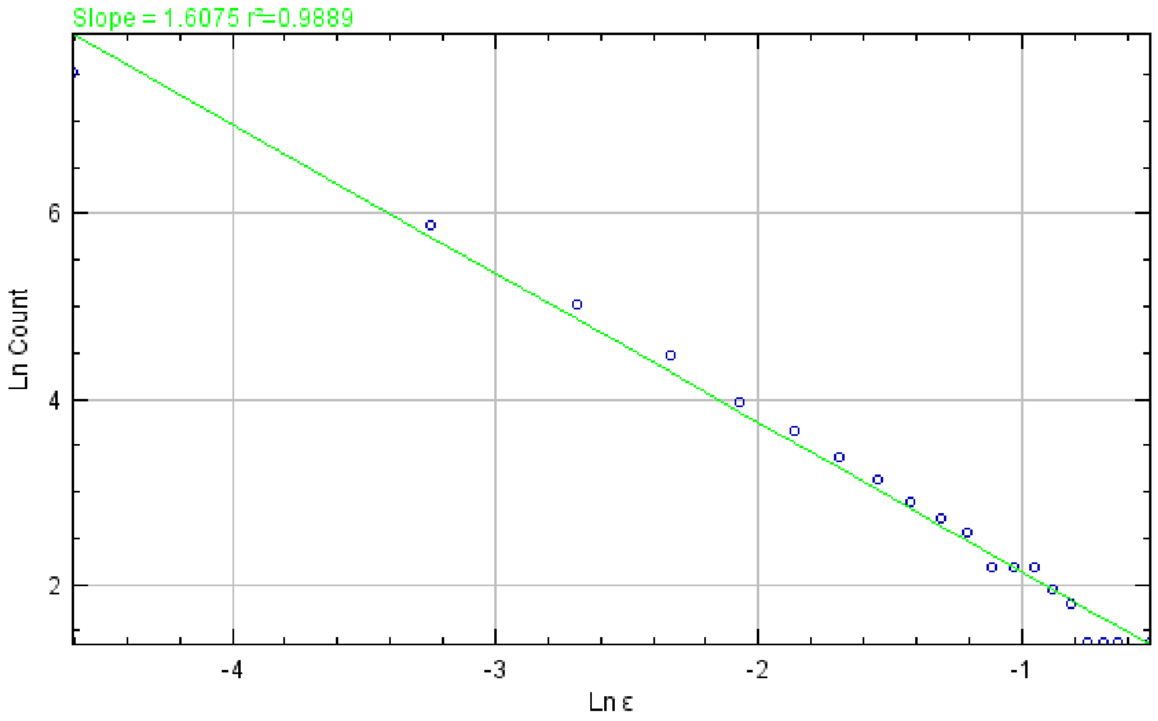
Slope = 1.6075 r²=0.9889
Ln Count
Ln ε
6
4
2
-4
-3
-2
-1

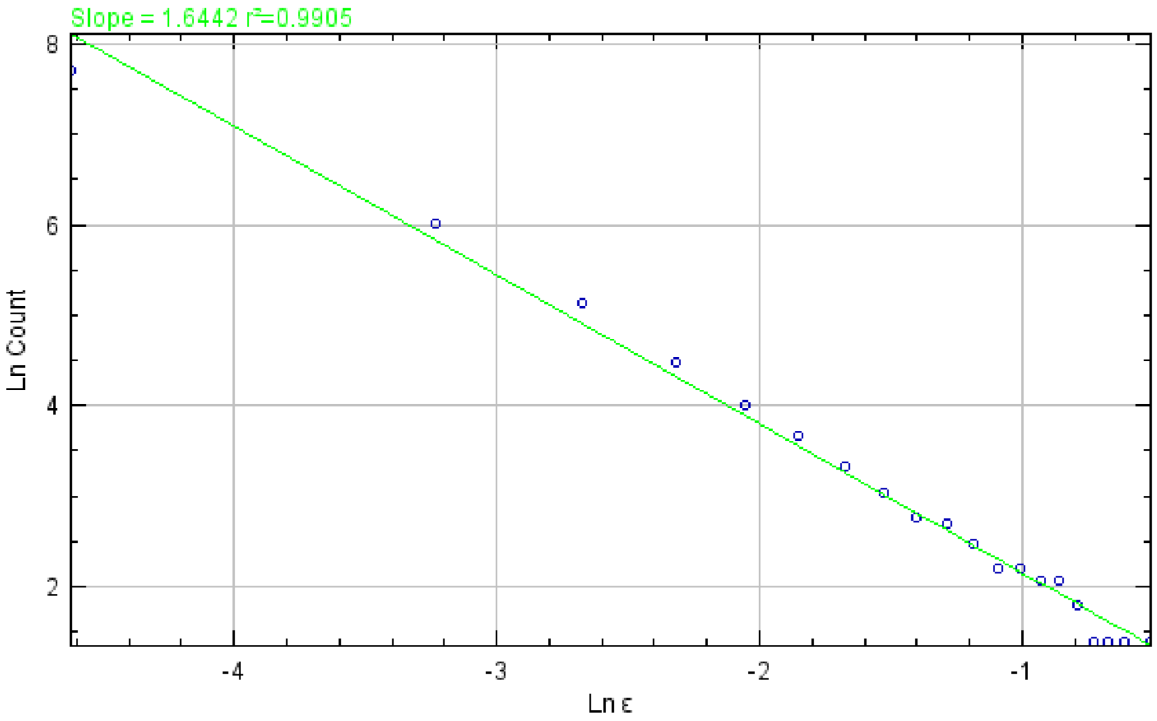
Slope = 1.6442 r²=0.9905
Ln Count
Ln ε
8
6
4
2
-4
-3
-2
-1

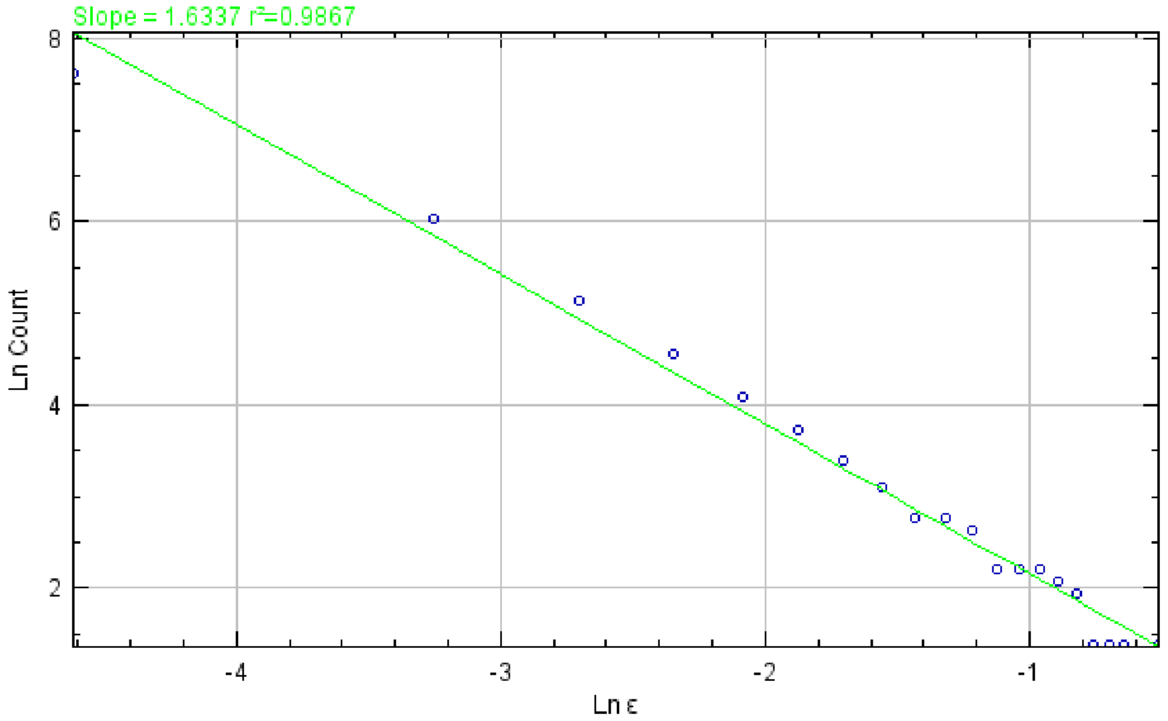
Slope = 1.6337 r²=0.9867
Ln Count
Ln ε
8
6
4
2
-4
-3
-2
-1

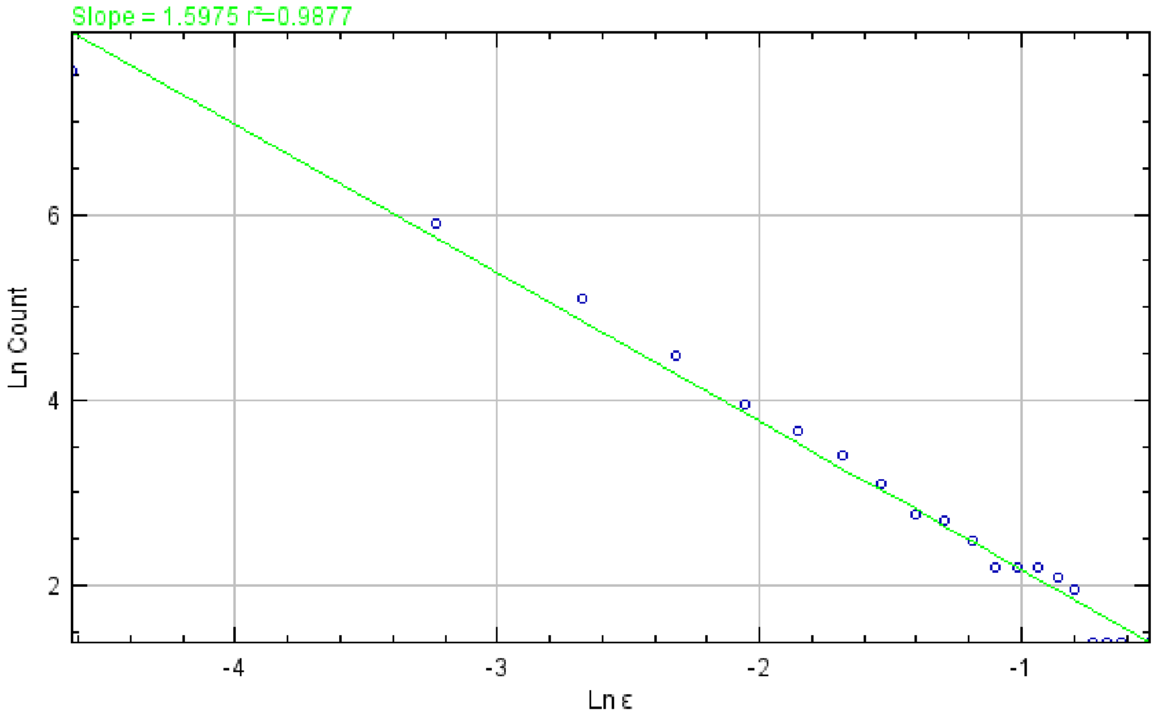
Slope = 1.5975 r²=0.9877
Ln Count
Ln ε
6
4
2
-4
-3
-2
-1

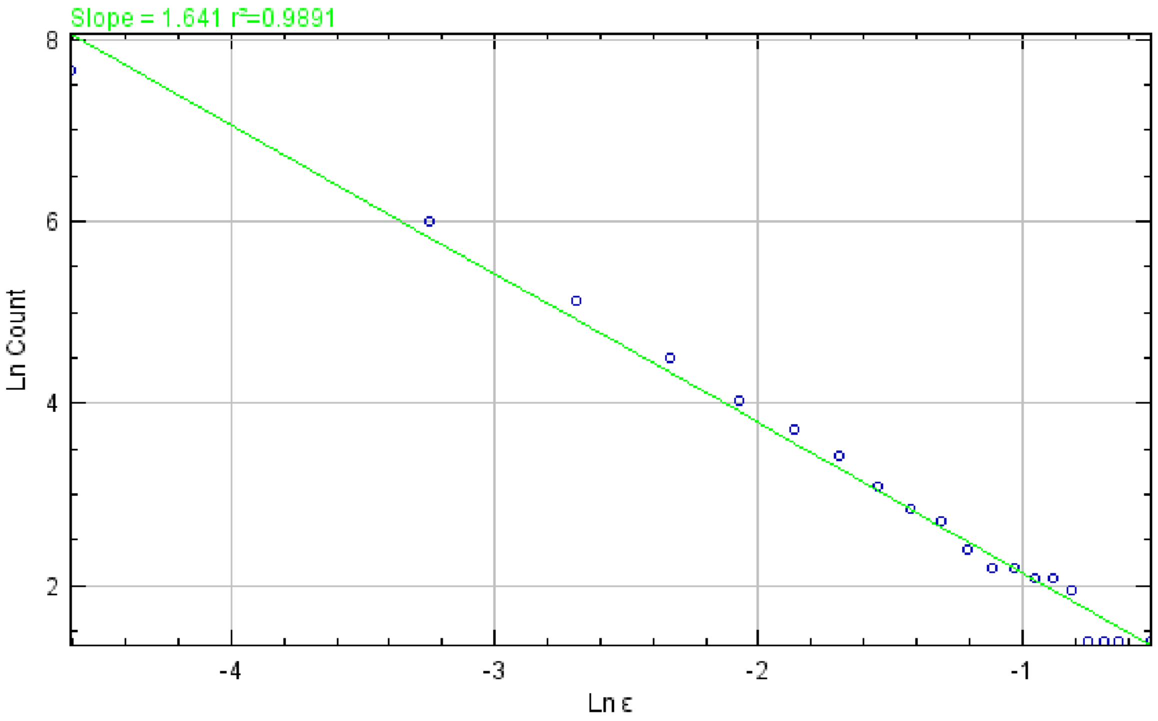
Slope = 1.641 r²=0.9891
Ln Count
Ln ε
8
6
4
2
-4
-3
-2
-1

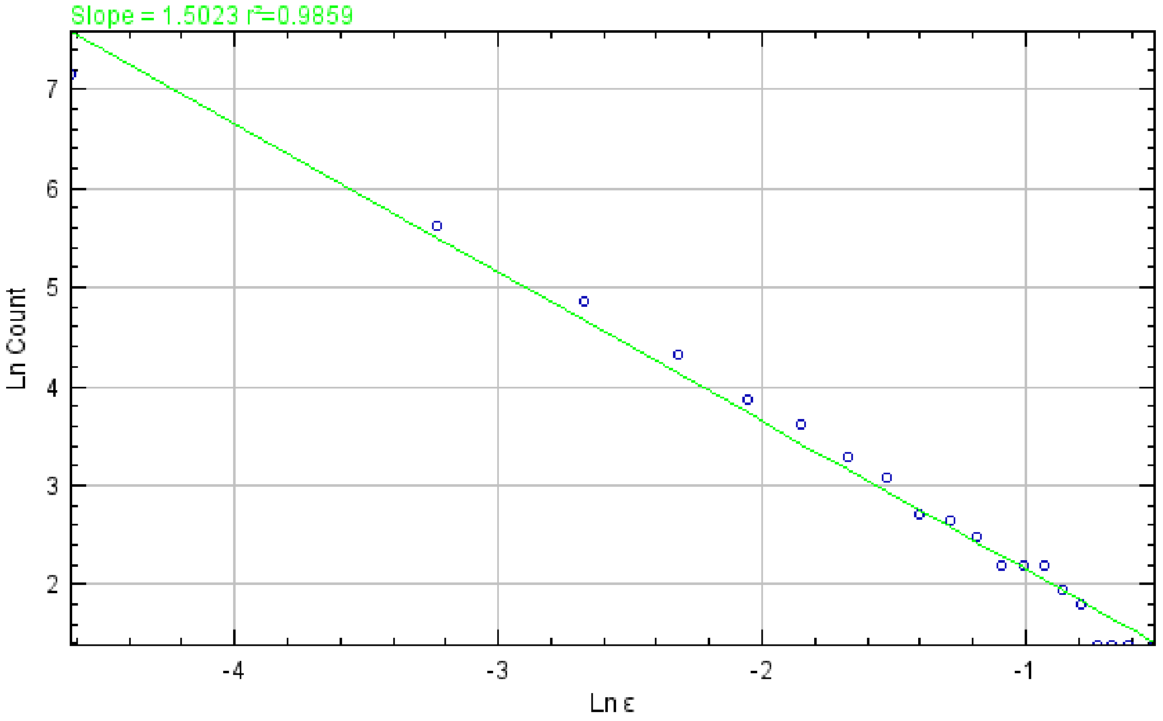
Slope = 1.5023 r²=0.9859
Ln Count
Ln ε
7
6
5
4
3
2
-4
-3
-2
-1

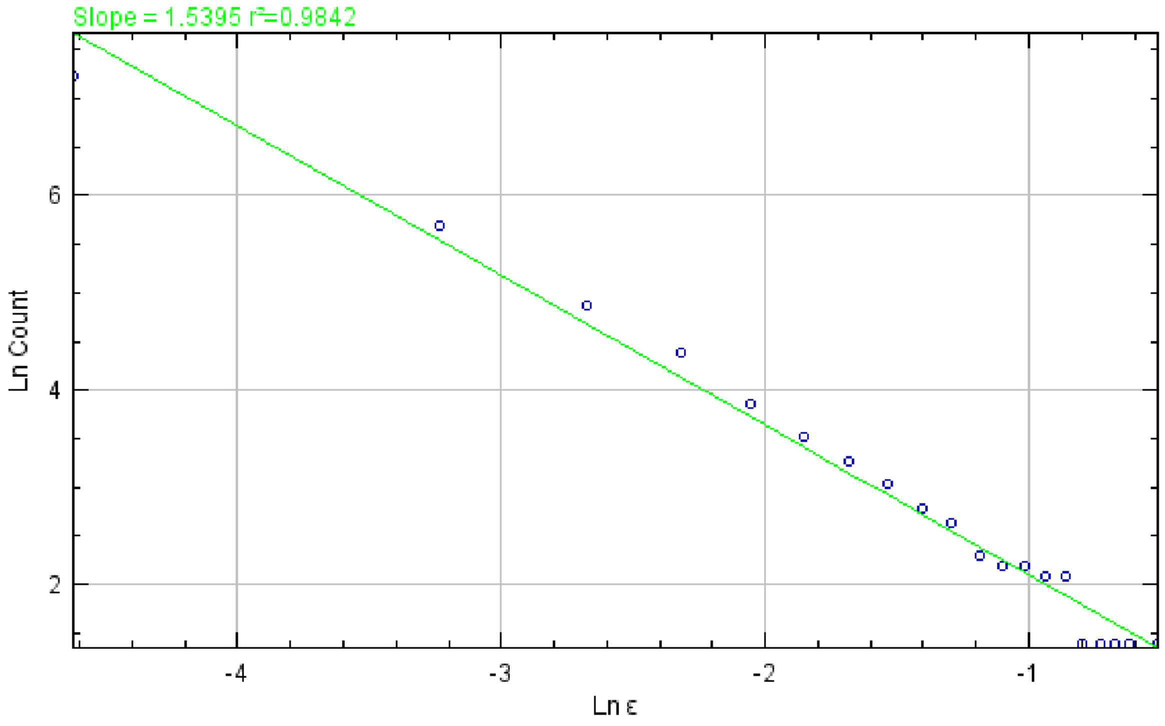

Slope = 1.5395 r²=0.9842
Ln Count
Ln ε


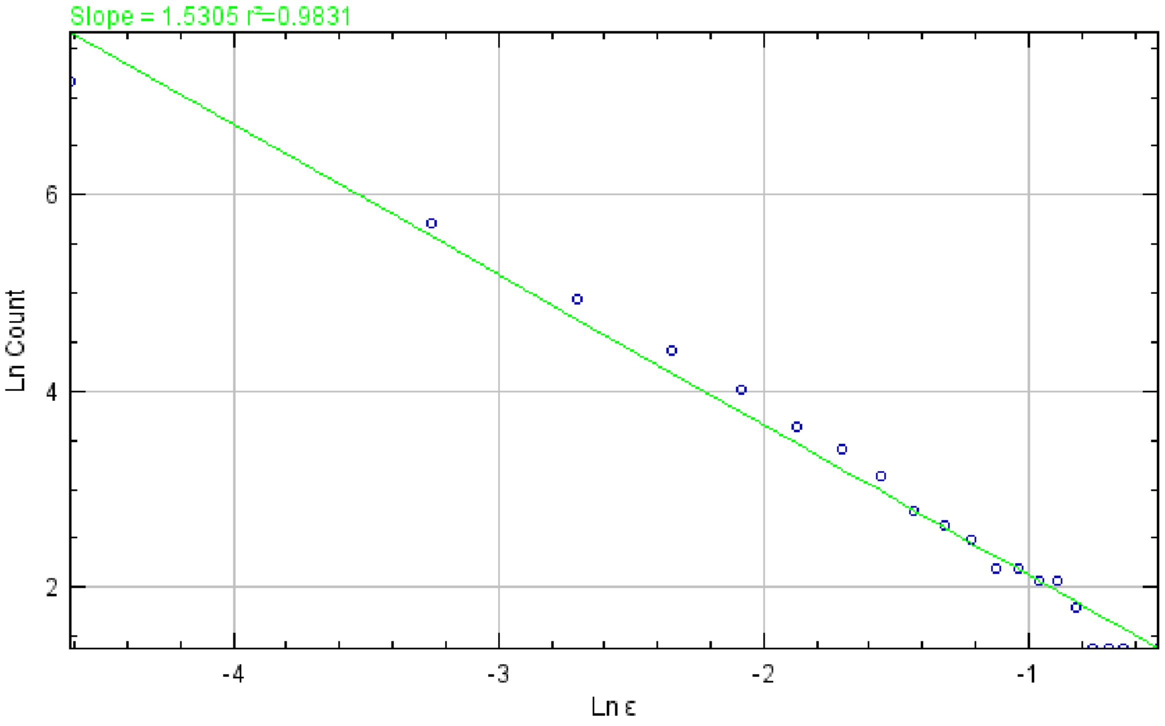

Slope = 1.5305 r²=0.9831
Ln Count
Ln ε

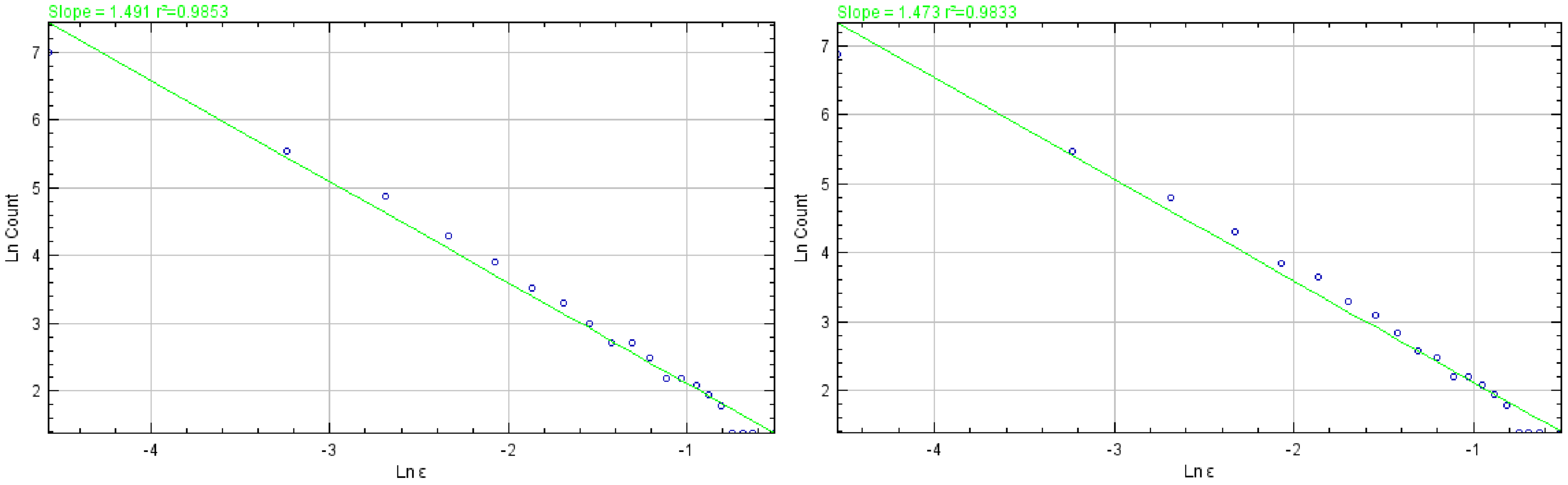
Slope = 1.491 r²=0.9853
Ln Count
Ln ε
Slope = 1.473 r²=0.9833
Ln Count
Ln ε

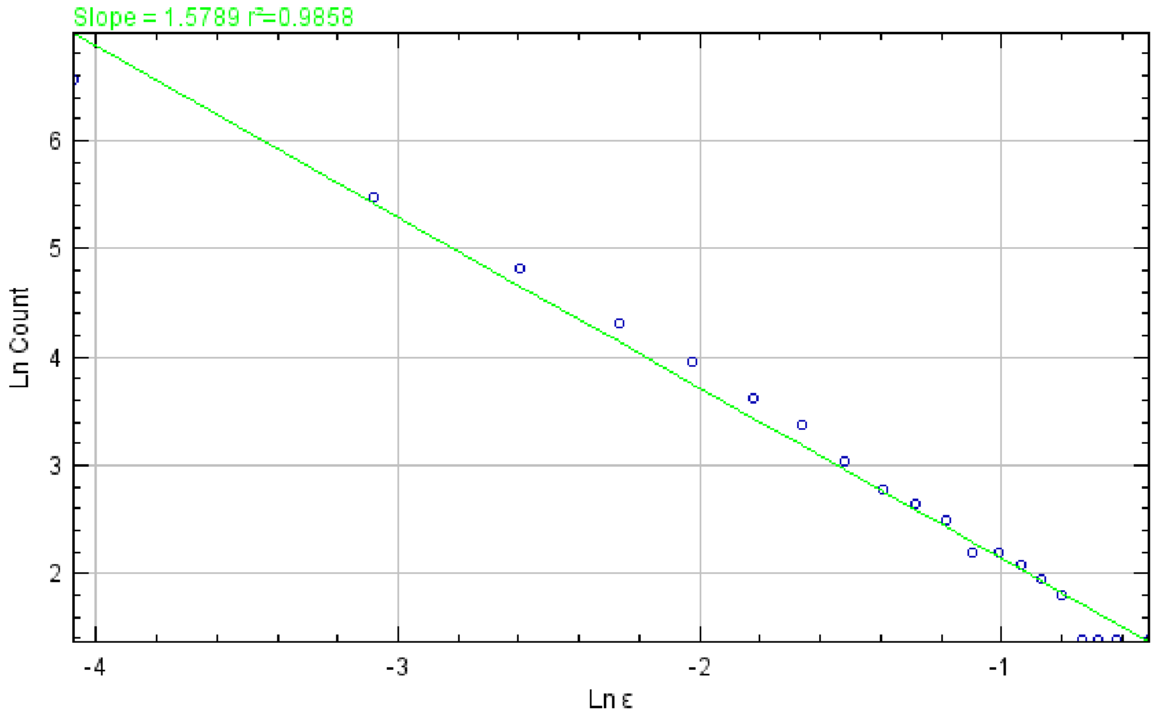
Slope = 1.5789 r²=0.9858
Ln Count
Ln ε

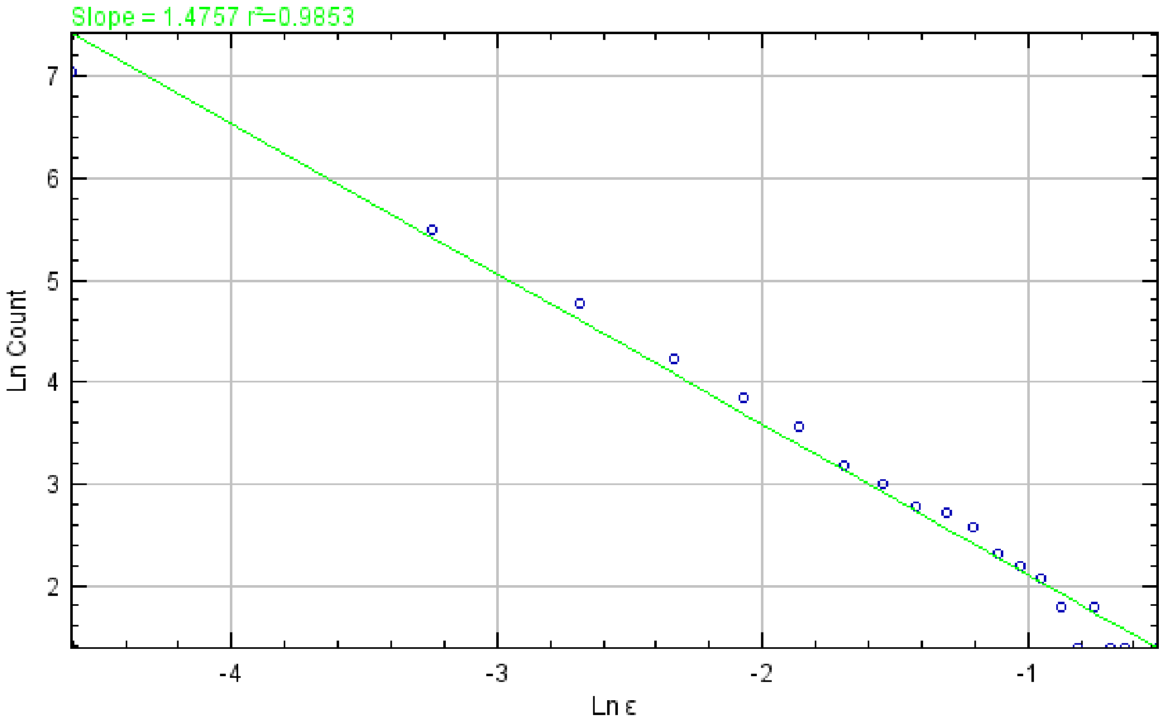
Slope = 1.4757 r²=0.9853
Ln Count
Ln ε

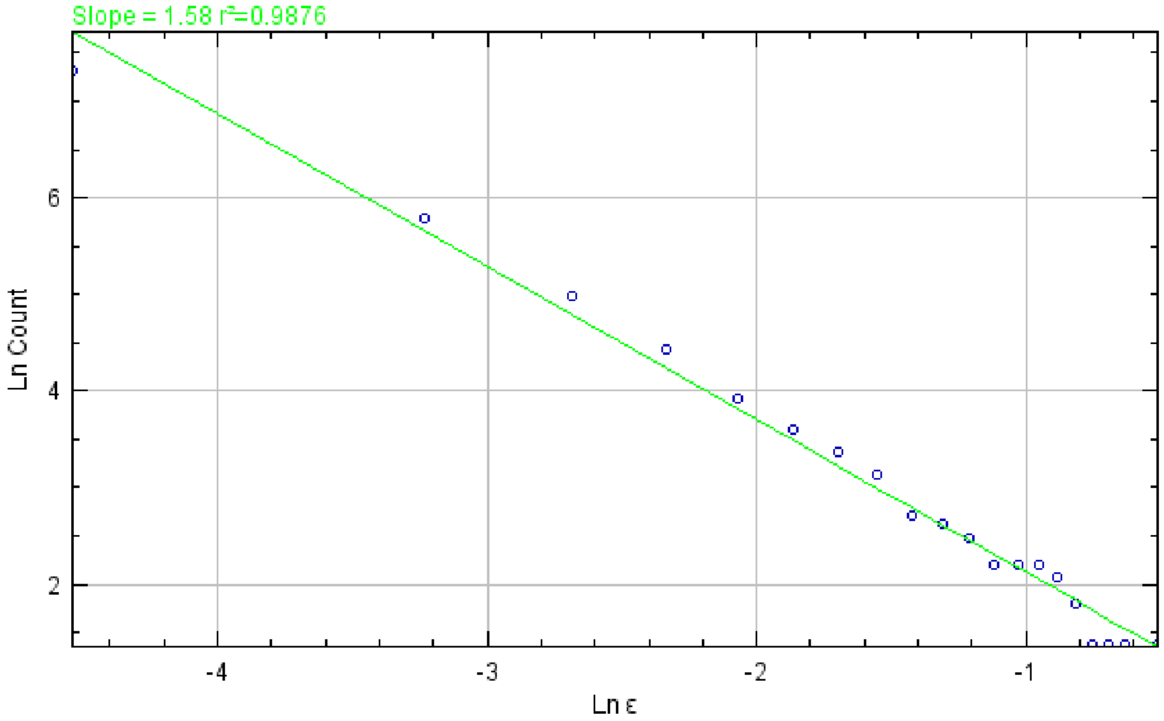
Slope = 1.58 r²=0.9876
Ln Count
6
4
2
-4
-3
-2
-1
Ln ε

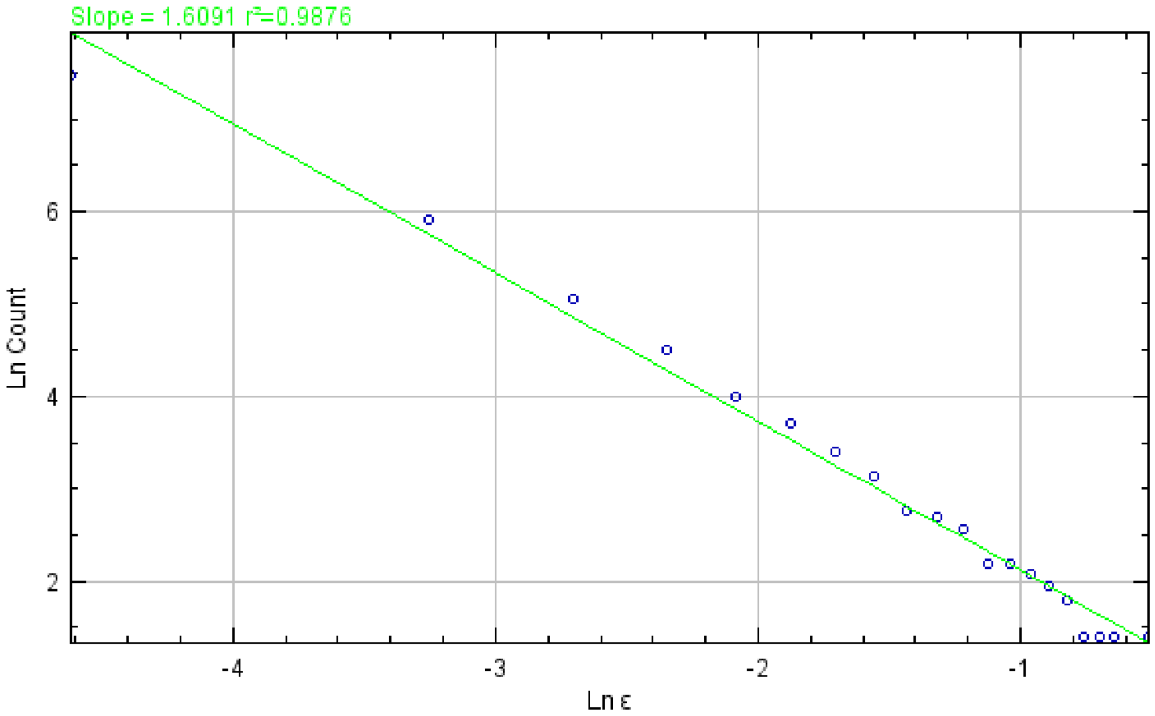
Slope = 1.6091 r²=0.9876
Ln Count
6
4
2
-4
-3
-2
-1
Ln ε
