# Supplementary material for: Investigating Multimodal Diagnostic Eye Biomarkers of Cognitive Impairment by Measuring Vascular and Neurogenic Changes in the Retina
Source: Front Physiol. 2018 Dec 6;9:1721. doi: 10.3389/fphys.2018.01721 (PMC6291749; doi:10.3389/fphys.2018.01721)
Supplement: Supplementary file 2 [file Data_Sheet_2.PDF]

Supplementary Information: Double log plots of the remaining cognitively healthy participants not shown in the manuscript. The subsequent pages contain a plot of the log of the number of boxes containing meaningful pixels vs. the log of the box sizes with their corresponding slope and R^2 values. The slope represents D_0 (counting box fractal dimension) and the R^2 values shows the quality of the regression plots. Each page contains plots for two subjects.

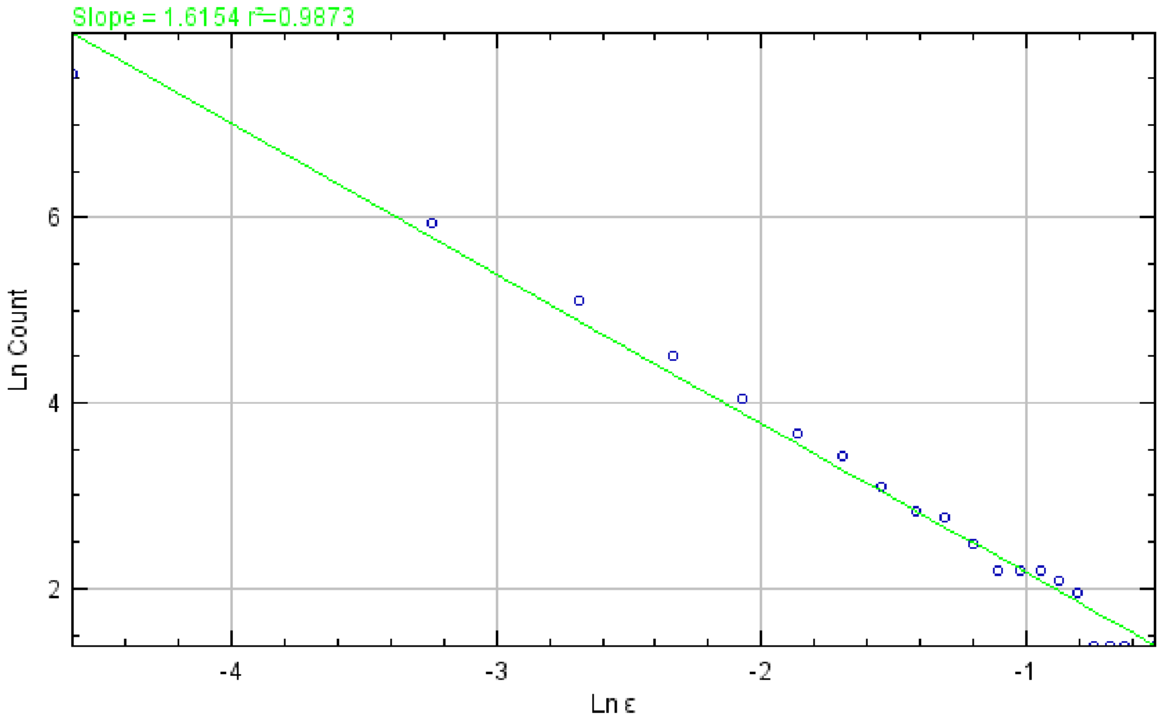
Slope = 1.6154 r²=0.9873
Ln Count
6
4
2
-4
-3
-2
-1
Ln ε

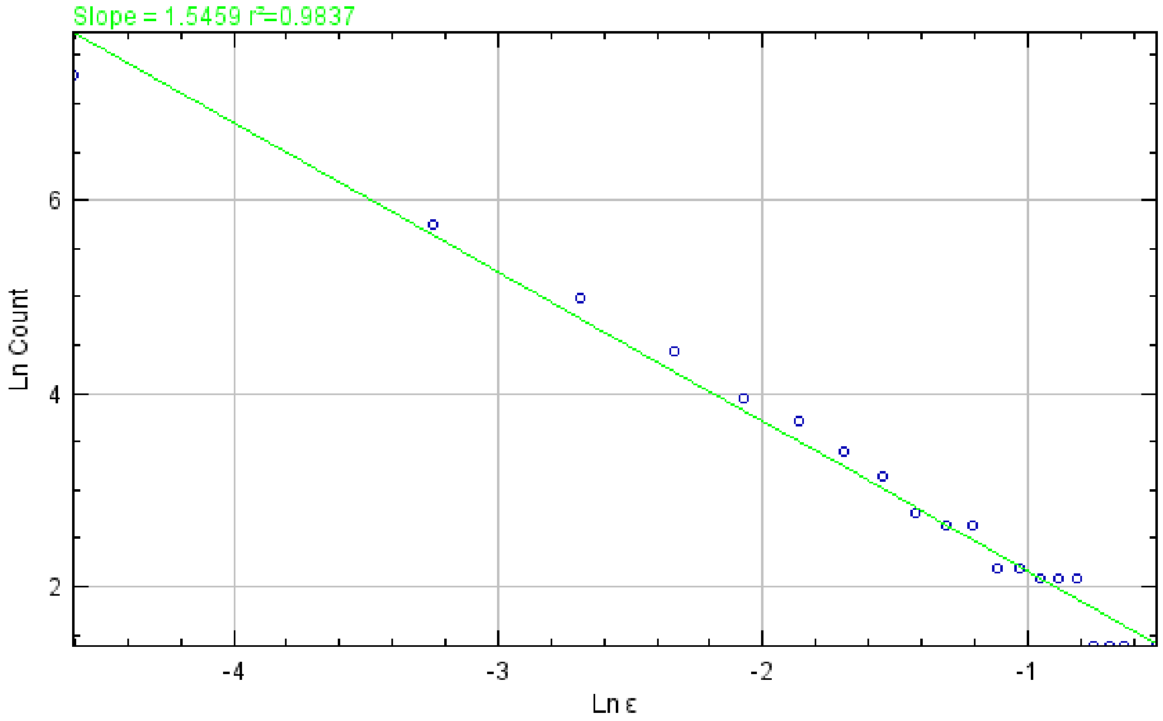
Slope = 1.5459 r²=0.9837
Ln Count
6
4
2
-4
-3
-2
-1
Ln ε

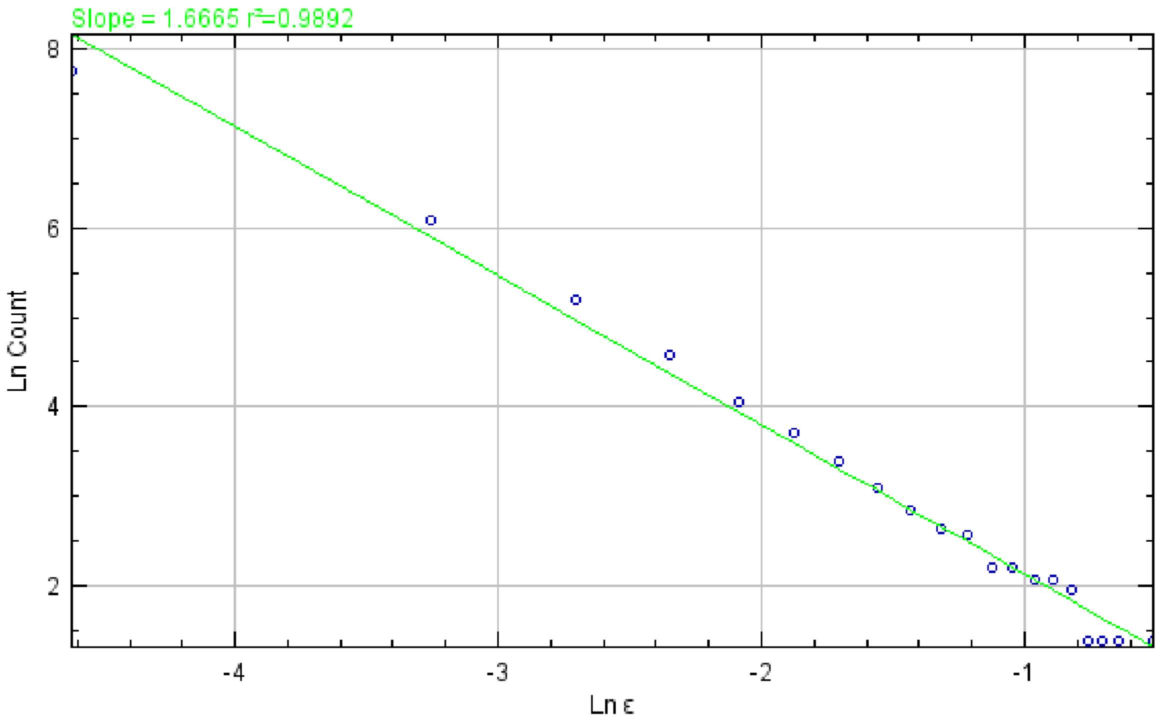

Slope = 1.6665 r²=0.9892
Ln Count
Ln ε

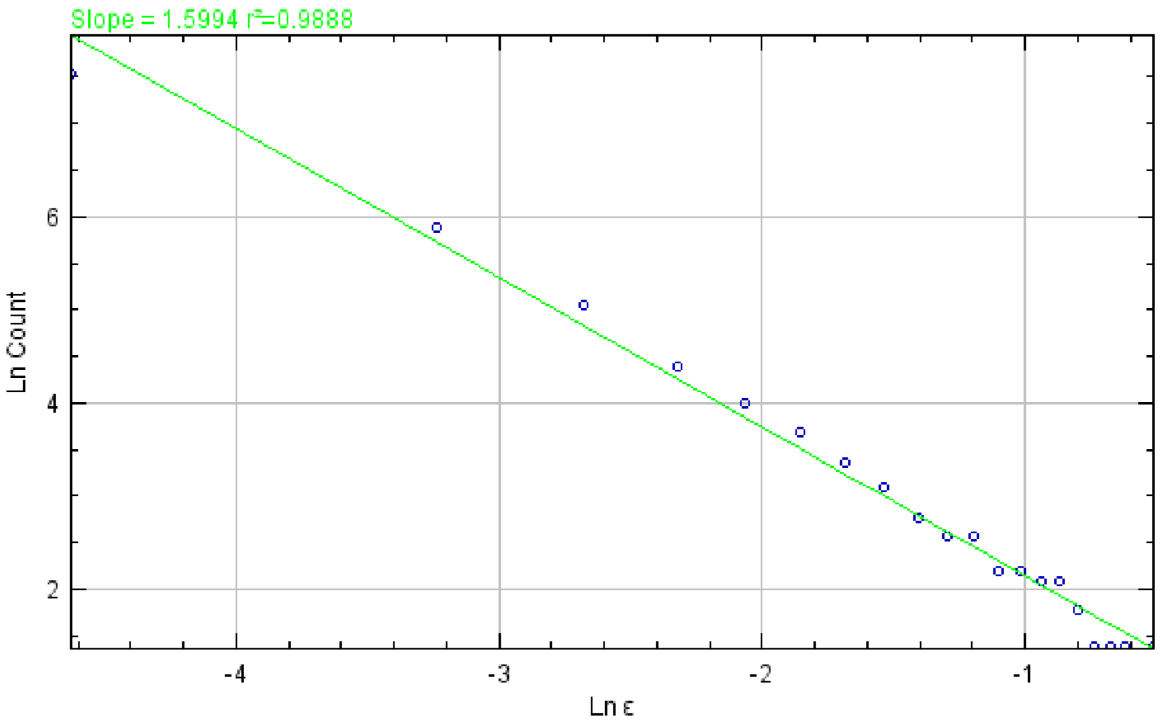

Slope = 1.5994 r²=0.9888
Ln Count
Ln ε

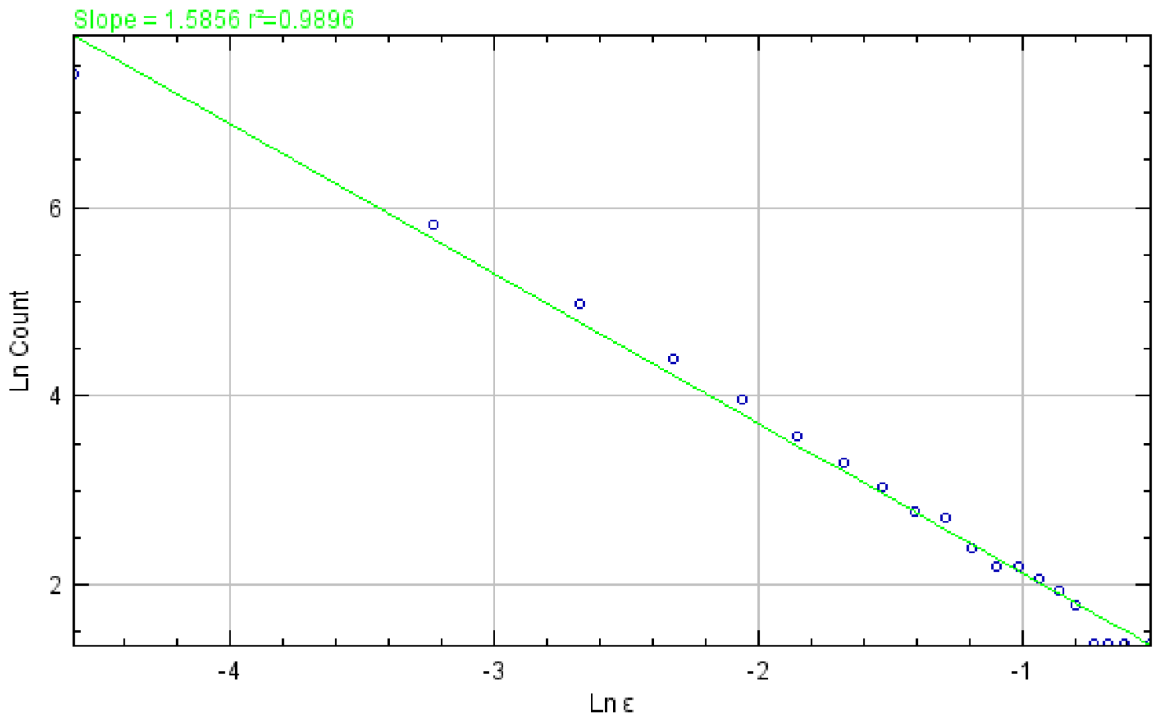
Slope = 1.5856 r²=0.9896
Ln Count
6
4
2
-4
-3
-2
-1
Ln ε

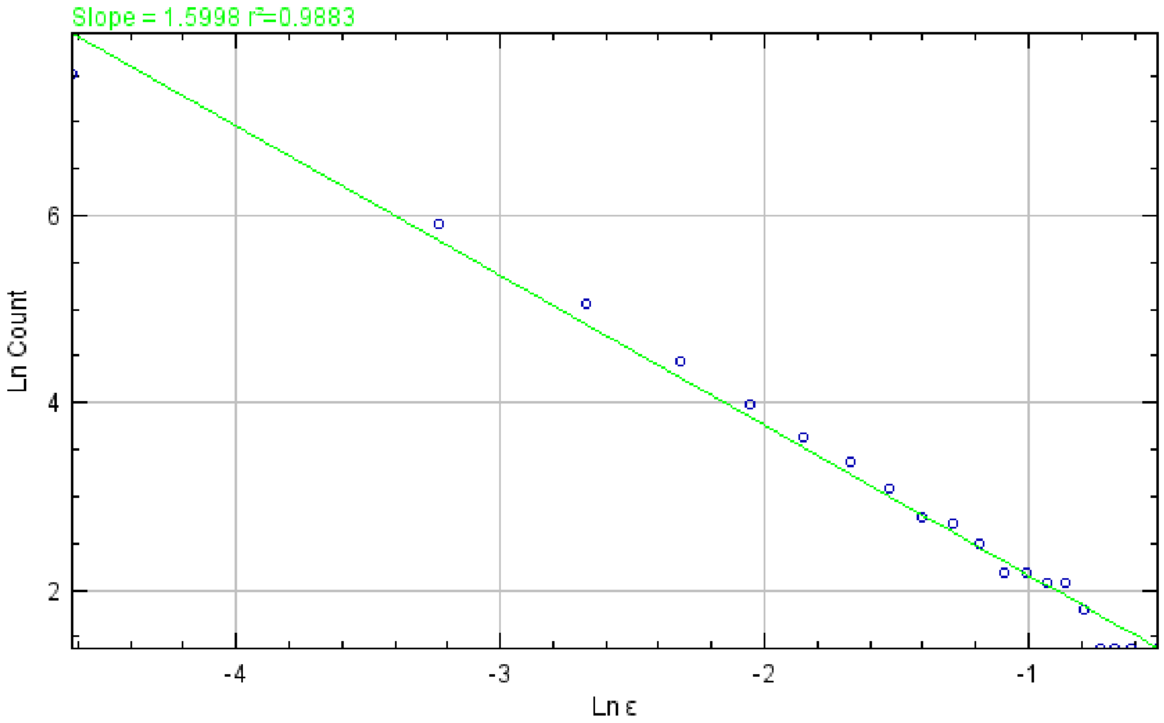
Slope = 1.5998 r²=0.9883
Ln Count
6
4
2
-4
-3
-2
-1
Ln ε

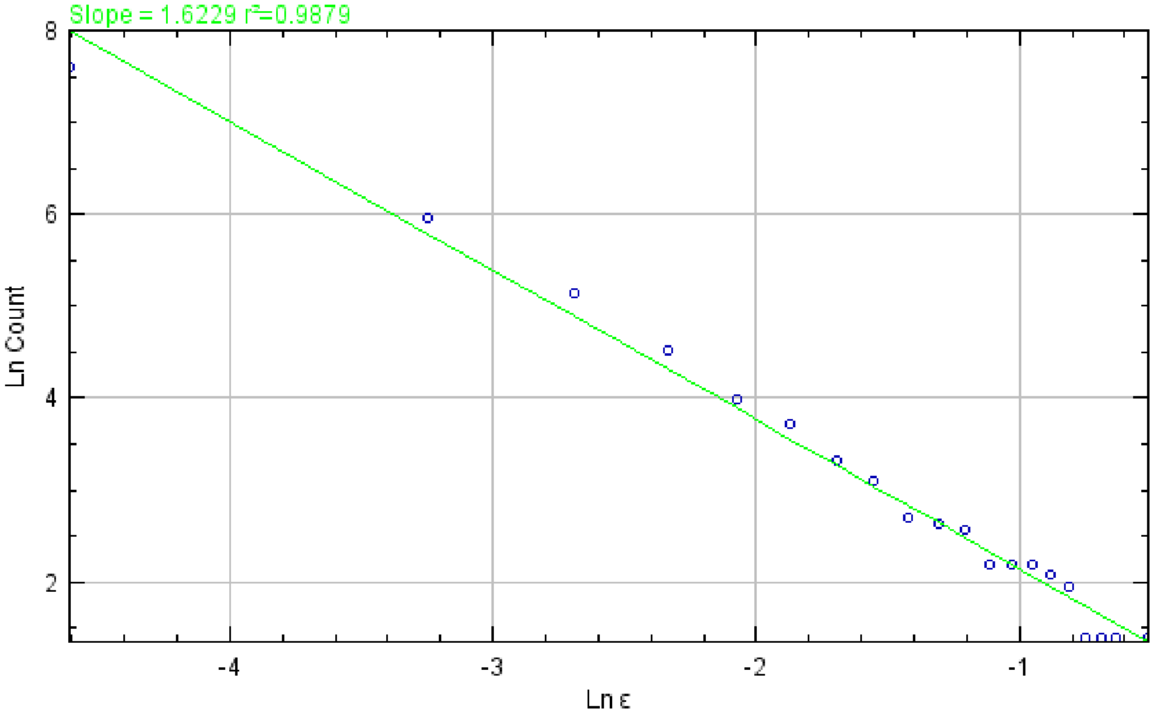
Slope = 1.6229 r²=0.9879
Ln Count
Ln ε
8
6
4
2
-4
-3
-2
-1

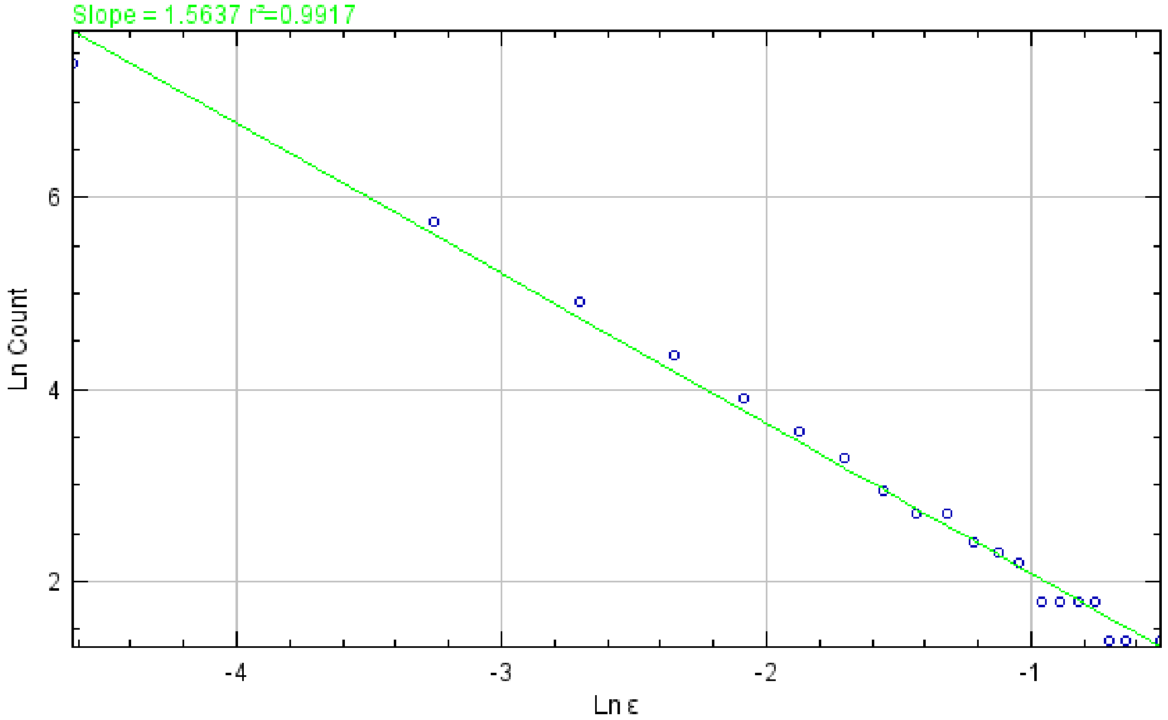
Slope = 1.5637 r²=0.9917
Ln Count
Ln ε
6
4
2
-4
-3
-2
-1

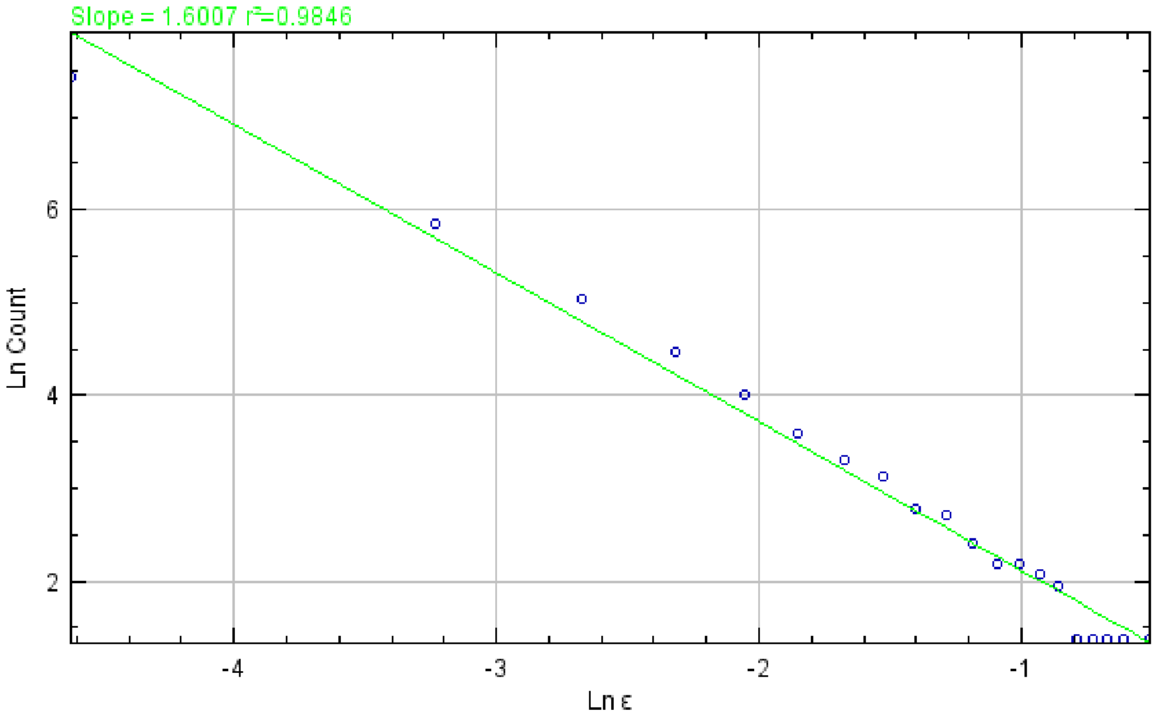
Slope = 1.6007 r²=0.9846
Ln Count
Ln ε
6
4
2
-4
-3
-2
-1

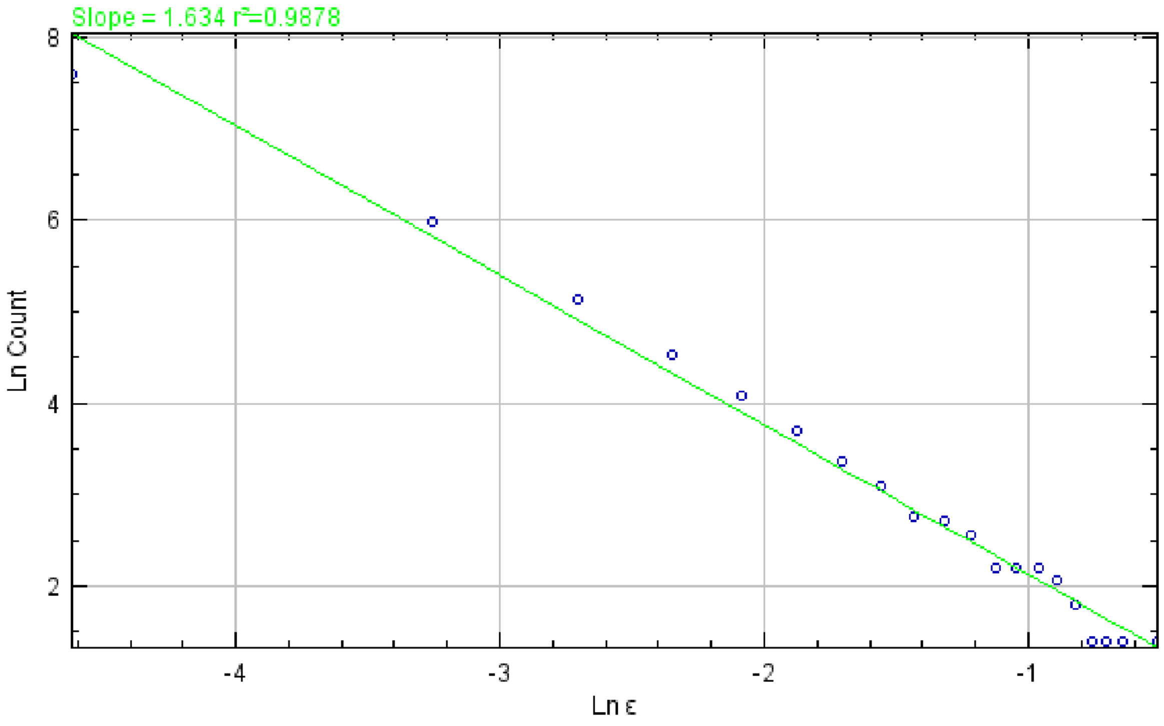
Slope = 1.634 r²=0.9878
Ln Count
Ln ε
8
6
4
2
-4
-3
-2
-1

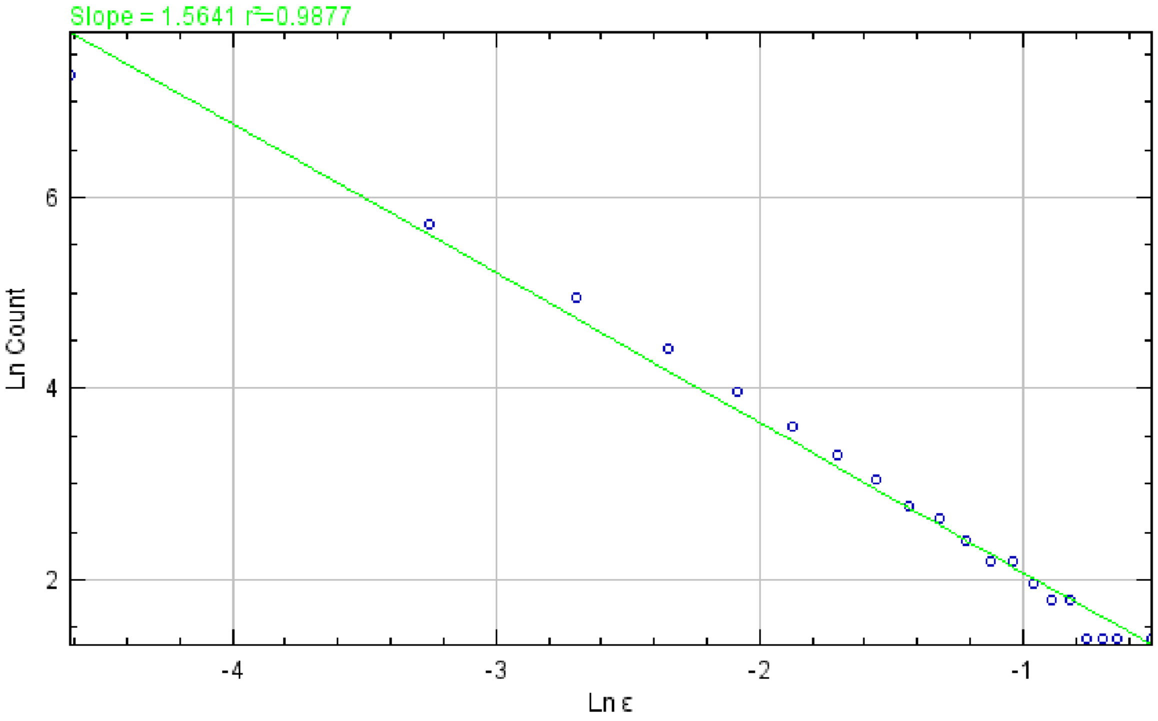
Slope = 1.5641 r²=0.9877
Ln Count
Ln ε
6
4
2
-4
-3
-2
-1

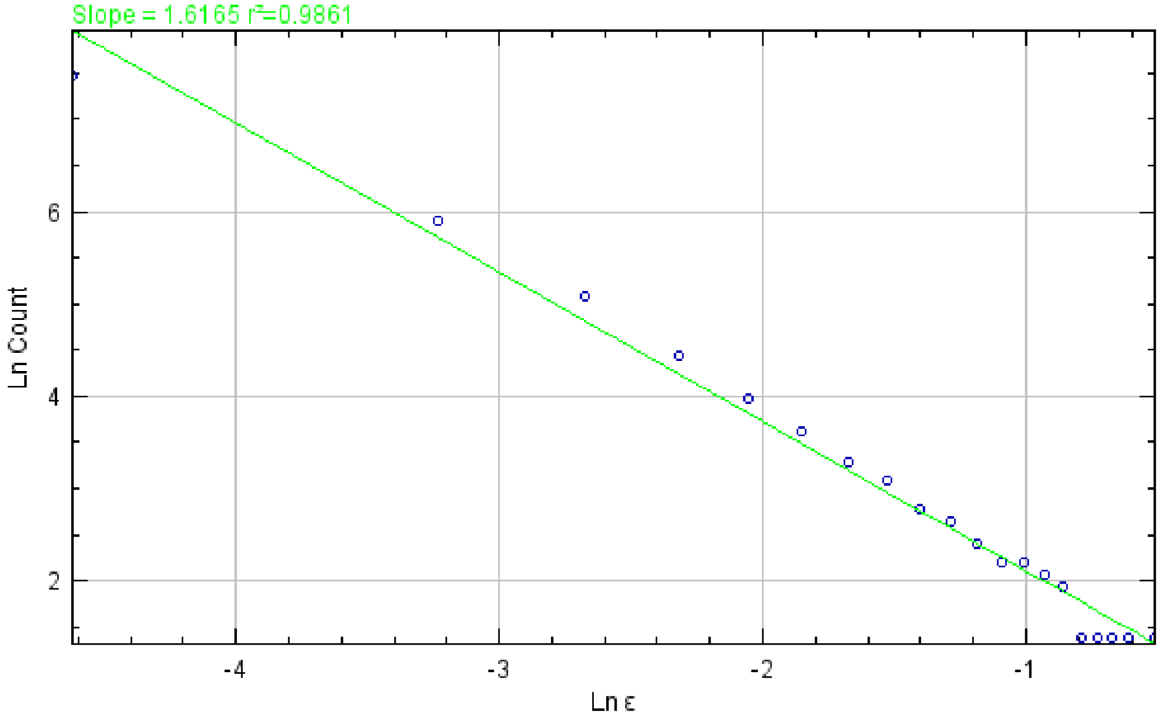
Slope = 1.6165 r²=0.9861
Ln Count
Ln ε
6
4
2
-4
-3
-2
-1

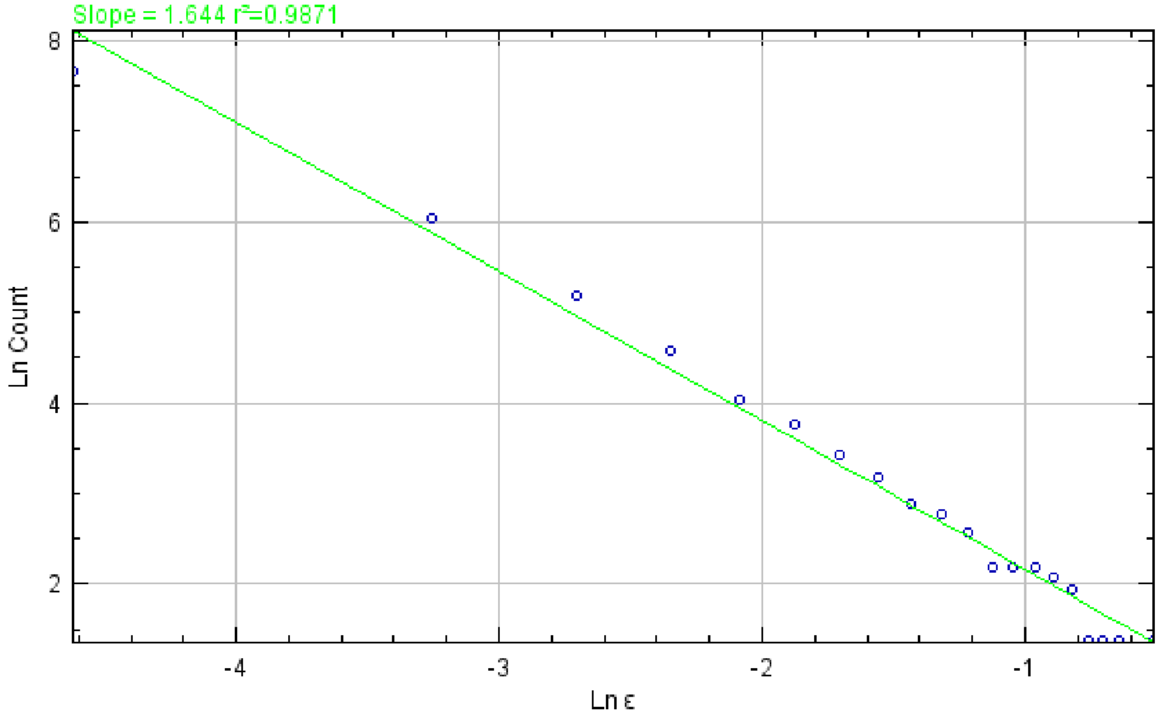

Slope = 1.644 r²=0.9871
Ln Count
Ln ε

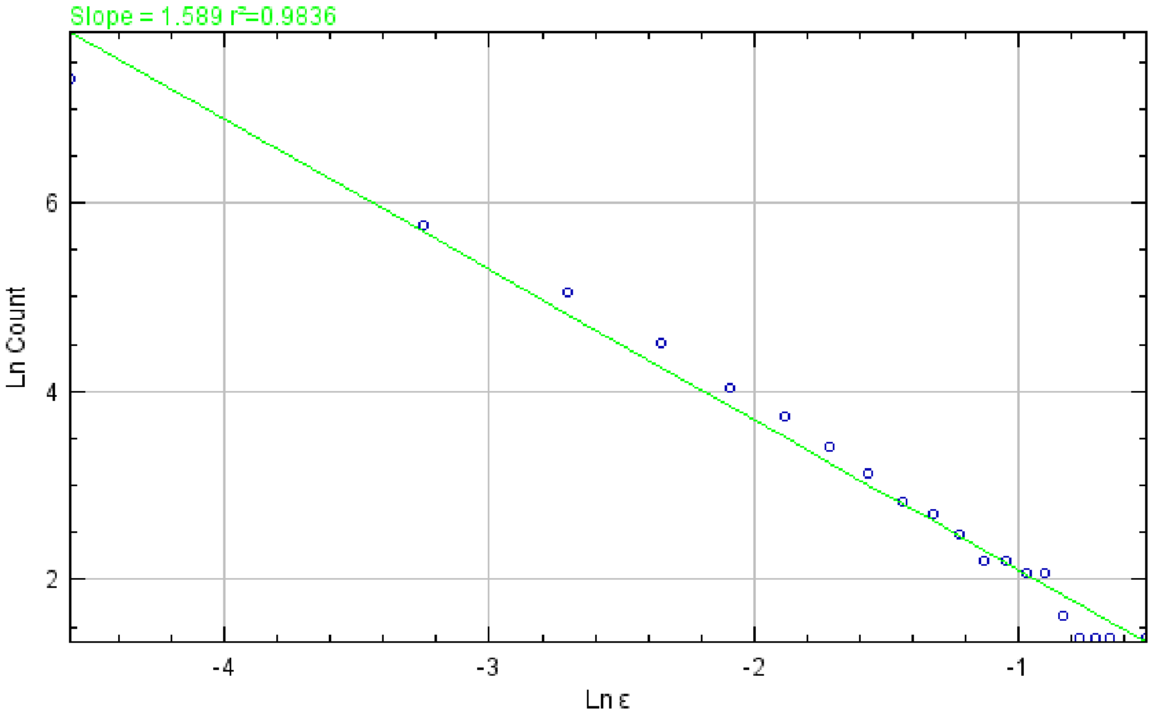

Slope = 1.589 r²=0.9836
Ln Count
Ln ε

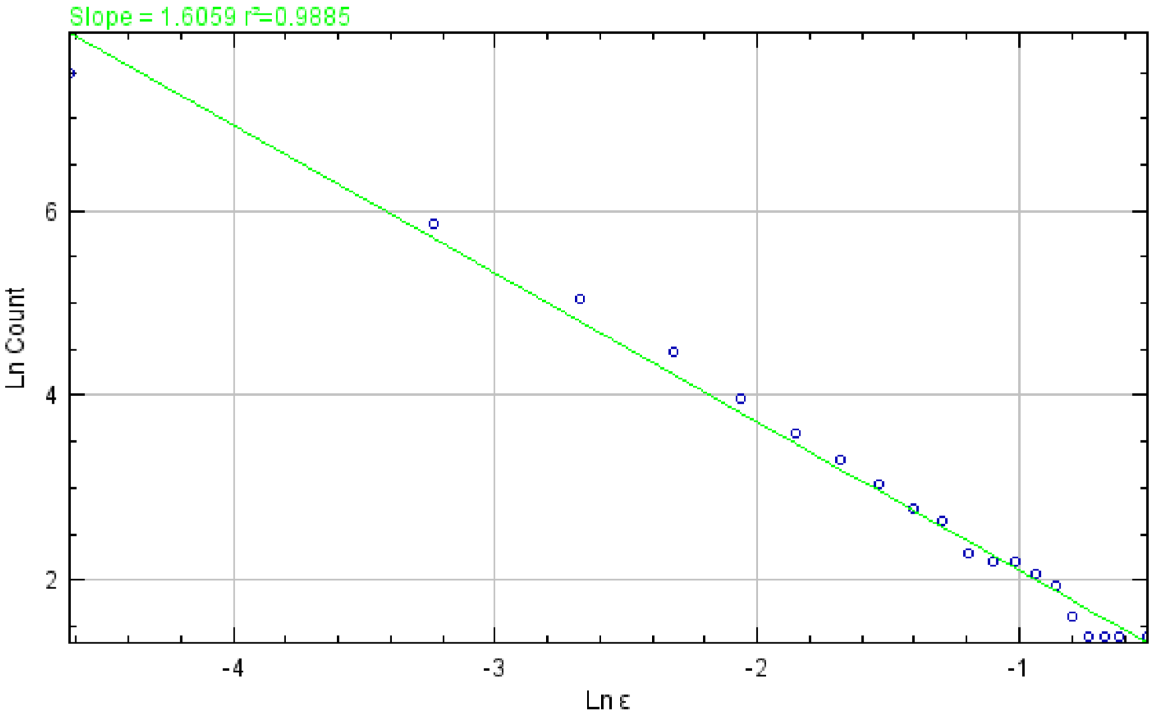

Slope = 1.6059 r²=0.9885
Ln Count
Ln ε

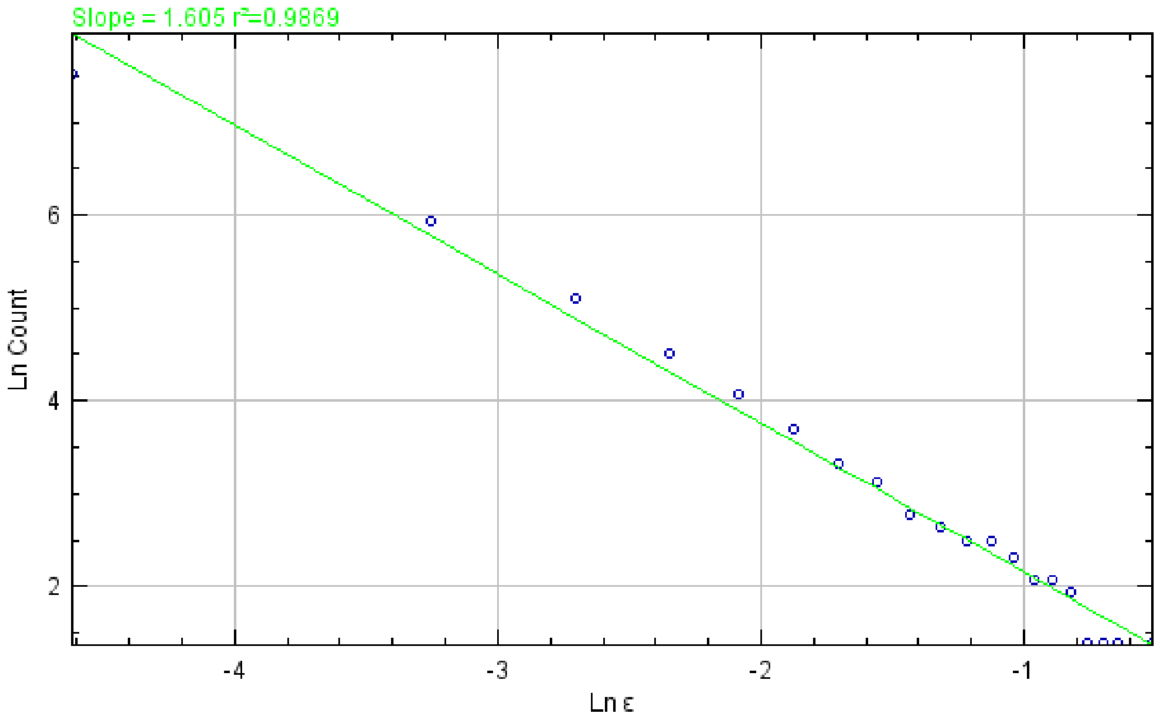

Slope = 1.605 r²=0.9869
Ln Count
Ln ε

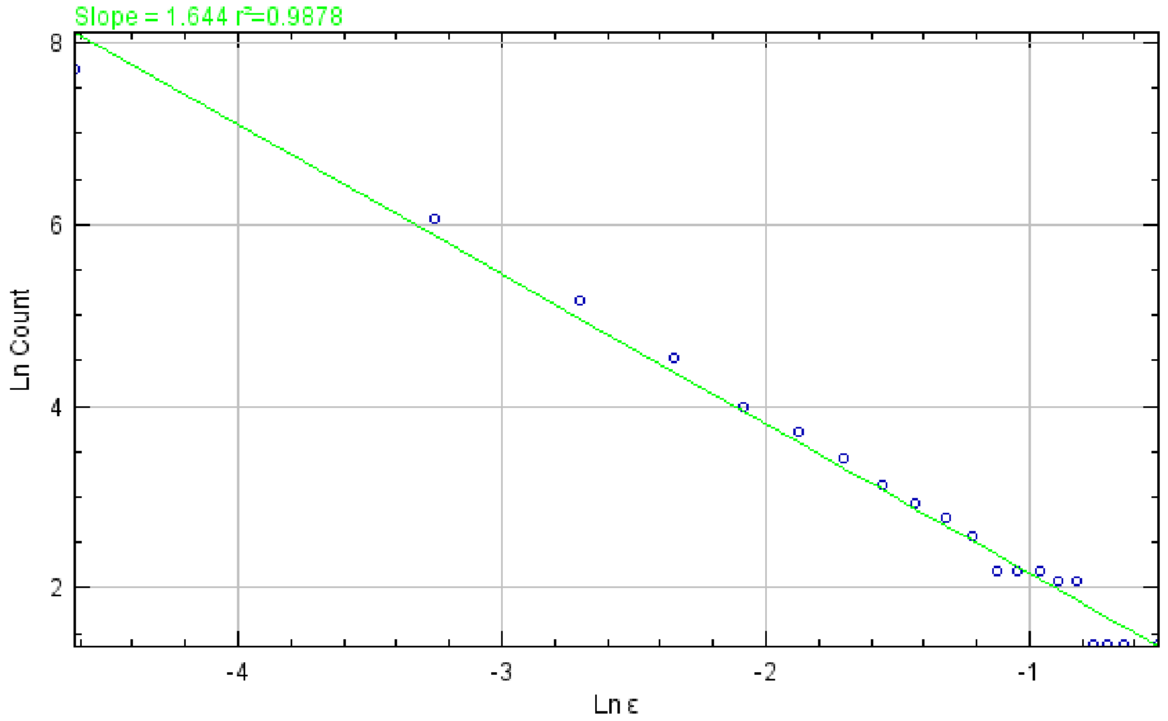
Slope = 1.644 r²=0.9878
8
6
4
2
Ln Count
-4
-3
-2
-1
Ln ε

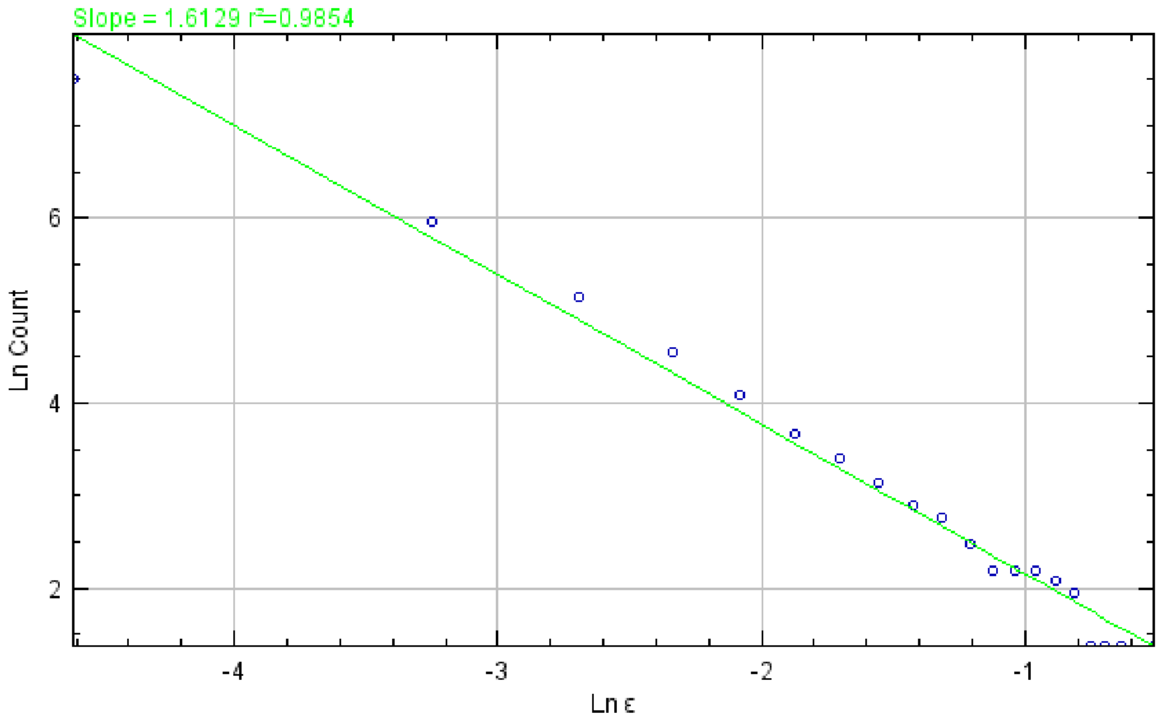
Slope = 1.6129 r²=0.9854
6
4
2
Ln Count
-4
-3
-2
-1
Ln ε
