# Supplementary material for: Investigating Multimodal Diagnostic Eye Biomarkers of Cognitive Impairment by Measuring Vascular and Neurogenic Changes in the Retina
Source: Front Physiol. 2018 Dec 6;9:1721. doi: 10.3389/fphys.2018.01721 (PMC6291749; doi:10.3389/fphys.2018.01721)
Supplement: Supplementary file 3 [file Data_Sheet_3.PDF]

Supplementary Material

Investigating Multimodal Diagnostic Eye Biomarkers of Cognitive Impairment by Measuring Vascular and Neurogenic Changes in The Retina

Delia Cabrera DeBuc[1*], Gabor Mark Somfai[2,3], Edmund Arthur[1], Maja Kostic[1], Susel Oropesa[1], Carlos Mendoza Santiesteban[1]

[1]Bascom Palmer Eye Institute, Department of Ophthalmology, University of Miami, Miami, FL, USA

[2]Retinology Unit, Pallas Kliniken, Olten, Switzerland

[3]Department of Ophthalmology, Semmelweis University, Budapest, Hungary

*** Correspondence:** Delia Cabrera DeBuc, Ph.D: dcabrera2@med.miami.edu

1 Supplementary Tables

Table 1. Pearson product moment correlations between variables

		Flicker Amp	Flicker ImplT	MoCA Score (using cutoff)	RED OD	GREEN OD	BLUE OD	Big6craeC	Big6crveC	Big6avrC	cTORTa	BCa	AFa	cTORTv	BCv
Flicker Amp	Pearson Correlation	1	.153	.347	-.089	-.164	-.073	-.243	.111	.166	-.138	.268	-.142	-.176	.217
	Sig. (2-tailed)		.520	.134	.708	.490	.759	.302	.643	.484	.561	.254	.549	.458	.359
	N	20	20	20	20	20	20	20	20	20	20	20	20	20	20
Flicker ImplT	Pearson Correlation	.153	1	.254	-.183	-.027	-.112	.144	-.176	.749**	-.267	-.378	.136	-.026	-.257
	Sig. (2-tailed)	.520		.280	.439	.909	.638	.544	.457	.000	.255	.100	.568	.914	.274
	N	20	20	20	20	20	20	20	20	20	20	20	20	20	20

MoCA Score (using cutoff)	Pearson Correlation	.347	.254	1	.347	.223	.100	-.265	.036	.289	-.377	-.219	.068	-.183	.307
	Sig. (2-tailed)	.134	.280		.134	.344	.676	.259	.879	.217	.101	.353	.776	.441	.188
	N	20	20	20	20	20	20	20	20	20	20	20	20	20	20
RED OD	Pearson Correlation	-.089	-.183	.347	1	.630**	.662**	-.303	-.107	-.306	-.033	-.213	-.285	-.209	.186
	Sig. (2-tailed)	.708	.439	.134		.003	.001	.194	.654	.189	.890	.367	.224	.377	.432
	N	20	20	20	20	20	20	20	20	20	20	20	20	20	20
GREEN OD	Pearson Correlation	-.164	-.027	.223	.630**	1	.625**	.200	-.049	-.069	-.136	-.410	-.322	-.006	.015
	Sig. (2-tailed)	.490	.909	.344	.003		.003	.398	.837	.772	.568	.073	.166	.979	.949
	N	20	20	20	20	20	20	20	20	20	20	20	20	20	20
BLUE OD	Pearson Correlation	-.073	-.112	.100	.662**	.625**	1	-.187	.106	-.075	.039	-.228	-.172	-.026	.173
	Sig. (2-tailed)	.759	.638	.676	.001	.003		.431	.657	.752	.871	.334	.469	.912	.466
	N	20	20	20	20	20	20	20	20	20	20	20	20	20	20
Big6craeC	Pearson Correlation	-.243	.144	-.265	-.303	.200	-.187	1	-.062	.203	-.066	-.312	.108	.341	-.520*
	Sig. (2-tailed)	.302	.544	.259	.194	.398	.431		.795	.391	.782	.181	.649	.142	.019
	N	20	20	20	20	20	20	20	20	20	20	20	20	20	20
Big6crveC	Pearson Correlation	.111	-.176	.036	-.107	-.049	.106	-.062	1	-.082	.310	.047	.022	.256	.074
	Sig. (2-tailed)	.643	.457	.879	.654	.837	.657	.795		.732	.183	.844	.925	.276	.757
	N	20	20	20	20	20	20	20	20	20	20	20	20	20	20
Big6avrC	Pearson Correlation	.166	.749**	.289	-.306	-.069	-.075	.203	-.082	1	-.397	-.517*	.213	.105	-.279

	Sig. (2-tailed)	.484	.000	.217	.189	.772	.752	.391	.732		.083	.019	.367	.660	.233
	N	20	20	20	20	20	20	20	20	20	20	20	20	20	20
cTORTa	Pearson Correlation	-.138	-.267	-.377	-.033	-.136	.039	-.066	.310	-.397	1	.333	-.261	.486*	.373
	Sig. (2-tailed)	.561	.255	.101	.890	.568	.871	.782	.183	.083		.152	.266	.030	.105
	N	20	20	20	20	20	20	20	20	20	20	20	20	20	20
BCa	Pearson Correlation	.268	-.378	-.219	-.213	-.410	-.228	-.312	.047	-.517*	.333	1	-.221	.032	.410
	Sig. (2-tailed)	.254	.100	.353	.367	.073	.334	.181	.844	.019	.152		.348	.894	.073
	N	20	20	20	20	20	20	20	20	20	20	20	20	20	20
AFa	Pearson Correlation	-.142	.136	.068	-.285	-.322	-.172	.108	.022	.213	-.261	-.221	1	-.047	-.425
	Sig. (2-tailed)	.549	.568	.776	.224	.166	.469	.649	.925	.367	.266	.348		.843	.062
	N	20	20	20	20	20	20	20	20	20	20	20	20	20	20
cTORTv	Pearson Correlation	-.176	-.026	-.183	-.209	-.006	-.026	.341	.256	.105	.486*	.032	-.047	1	.161
	Sig. (2-tailed)	.458	.914	.441	.377	.979	.912	.142	.276	.660	.030	.894	.843		.497
	N	20	20	20	20	20	20	20	20	20	20	20	20	20	20
BCv	Pearson Correlation	.217	-.257	.307	.186	.015	.173	-.520*	.074	-.279	.373	.410	-.425	.161	1
	Sig. (2-tailed)	.359	.274	.188	.432	.949	.466	.019	.757	.233	.105	.073	.062	.497	
	N	20	20	20	20	20	20	20	20	20	20	20	20	20	20
AFv	Pearson Correlation	-.204	-.401	-.052	.110	.171	.233	.027	-.016	-.412	.324	.299	-.285	.211	.556*
	Sig. (2-tailed)	.389	.080	.829	.645	.472	.322	.911	.946	.071	.164	.200	.223	.373	.011
	N	20	20	20	20	20	20	20	20	20	20	20	20	20	20

cTORTt	Pearson Correlation	-.211	-.139	-.294	-.141	-.050	.031	.207	.327	-.105	.803**	.118	-.131	.905**	.287
	Sig. (2-tailed)	.373	.558	.208	.552	.835	.898	.381	.160	.661	.000	.620	.582	.000	.220
	N	20	20	20	20	20	20	20	20	20	20	20	20	20	20
BCt	Pearson Correlation	.205	-.340	-.090	.062	-.247	-.074	-.533*	.022	-.522*	.463*	.903**	-.364	.068	.596**
	Sig. (2-tailed)	.387	.143	.706	.797	.295	.758	.016	.928	.018	.040	.000	.114	.776	.006
	N	20	20	20	20	20	20	20	20	20	20	20	20	20	20
AFt	Pearson Correlation	-.392	-.190	-.175	-.058	-.068	-.083	.022	-.275	-.341	-.092	.126	.589**	-.270	-.296
	Sig. (2-tailed)	.087	.422	.461	.808	.776	.727	.927	.241	.141	.699	.595	.006	.250	.205
	N	20	20	20	20	20	20	20	20	20	20	20	20	20	20
LDRt	Pearson Correlation	.070	.475*	.156	-.056	-.403	-.403	-.227	-.129	.475*	-.162	-.276	.154	-.078	-.263
	Sig. (2-tailed)	.771	.034	.511	.815	.078	.078	.335	.586	.034	.494	.239	.517	.742	.263
	N	20	20	20	20	20	20	20	20	20	20	20	20	20	20
Do	Pearson Correlation	.384	.644**	.429	-.143	-.321	-.130	-.188	.028	.725**	-.086	-.210	.328	-.004	-.009
	Sig. (2-tailed)	.095	.002	.059	.548	.167	.584	.427	.908	.000	.719	.373	.158	.987	.969
	N	20	20	20	20	20	20	20	20	20	20	20	20	20	20
D1	Pearson Correlation	.353	.670**	.425	-.146	-.309	-.134	-.160	.011	.751**	-.094	-.248	.307	-.002	-.012
	Sig. (2-tailed)	.126	.001	.062	.539	.185	.572	.501	.964	.000	.694	.291	.188	.993	.958
	N	20	20	20	20	20	20	20	20	20	20	20	20	20	20
D2	Pearson Correlation	.322	.690**	.423	-.148	-.289	-.132	-.133	-.008	.772**	-.102	-.285	.292	-.001	-.020

	Sig. (2-tailed)	.166	.001	.063	.534	.216	.579	.575	.974	.000	.669	.223	.212	.997	.932
	N	20	20	20	20	20	20	20	20	20	20	20	20	20	20

Correlations

		AFv	cTORTt	BCt	AFt	LDRt	Do	D1	D2
Flicker Amp	Pearson Correlation	-.204	-.211	.205	-.392	.070	.384	.353	.322
	Sig. (2-tailed)	.389	.373	.387	.087	.771	.095	.126	.166
	N	20	20	20	20	20	20	20	20
Flicker ImplT	Pearson Correlation	-.401	-.139	-.340	-.190	.475*	.644**	.670**	.690**
	Sig. (2-tailed)	.080	.558	.143	.422	.034	.002	.001	.001
	N	20	20	20	20	20	20	20	20
MoCA Score (using cutoff)	Pearson Correlation	-.052	-.294	-.090	-.175	.156	.429	.425	.423
	Sig. (2-tailed)	.829	.208	.706	.461	.511	.059	.062	.063
	N	20	20	20	20	20	20	20	20
RED OD	Pearson Correlation	.110	-.141	.062	-.058	-.056	-.143	-.146	-.148
	Sig. (2-tailed)	.645	.552	.797	.808	.815	.548	.539	.534
	N	20	20	20	20	20	20	20	20
GREEN OD	Pearson Correlation	.171	-.050	-.247	-.068	-.403	-.321	-.309	-.289
	Sig. (2-tailed)	.472	.835	.295	.776	.078	.167	.185	.216
	N	20	20	20	20	20	20	20	20
BLUE OD	Pearson Correlation	.233	.031	-.074	-.083	-.403	-.130	-.134	-.132
	Sig. (2-tailed)	.322	.898	.758	.727	.078	.584	.572	.579
	N	20	20	20	20	20	20	20	20
Big6craeC	Pearson Correlation	.027	.207	-.533*	.022	-.227	-.188	-.160	-.133
	Sig. (2-tailed)	.911	.381	.016	.927	.335	.427	.501	.575
	N	20	20	20	20	20	20	20	20
Big6crveC	Pearson Correlation	-.016	.327	.022	-.275	-.129	.028	.011	-.008
	Sig. (2-tailed)	.946	.160	.928	.241	.586	.908	.964	.974
	N	20	20	20	20	20	20	20	20

Big6avrC	Pearson Correlation	-.412	-.105	-.522*	-.341	.475*	.725**	.751**	.772**
	Sig. (2-tailed)	.071	.661	.018	.141	.034	.000	.000	.000
	N	20	20	20	20	20	20	20	20
cTORTa	Pearson Correlation	.324	.803**	.463*	-.092	-.162	-.086	-.094	-.102
	Sig. (2-tailed)	.164	.000	.040	.699	.494	.719	.694	.669
	N	20	20	20	20	20	20	20	20
BCa	Pearson Correlation	.299	.118	.903**	.126	-.276	-.210	-.248	-.285
	Sig. (2-tailed)	.200	.620	.000	.595	.239	.373	.291	.223
	N	20	20	20	20	20	20	20	20
AFa	Pearson Correlation	-.285	-.131	-.364	.589**	.154	.328	.307	.292
	Sig. (2-tailed)	.223	.582	.114	.006	.517	.158	.188	.212
	N	20	20	20	20	20	20	20	20
cTORTv	Pearson Correlation	.211	.905**	.068	-.270	-.078	-.004	-.002	-.001
	Sig. (2-tailed)	.373	.000	.776	.250	.742	.987	.993	.997
	N	20	20	20	20	20	20	20	20
BCv	Pearson Correlation	.556*	.287	.596**	-.296	-.263	-.009	-.012	-.020
	Sig. (2-tailed)	.011	.220	.006	.205	.263	.969	.958	.932
	N	20	20	20	20	20	20	20	20
AFv	Pearson Correlation	1	.316	.257	.216	-.677**	-.295	-.306	-.307
	Sig. (2-tailed)		.174	.275	.361	.001	.207	.190	.187
	N	20	20	20	20	20	20	20	20
cTORTt	Pearson Correlation	.316	1	.211	-.212	-.135	-.037	-.039	-.041
	Sig. (2-tailed)	.174		.372	.369	.571	.876	.870	.865
	N	20	20	20	20	20	20	20	20
BCt	Pearson Correlation	.257	.211	1	-.004	-.146	-.145	-.177	-.210
	Sig. (2-tailed)	.275	.372		.986	.540	.541	.455	.374
	N	20	20	20	20	20	20	20	20
AFt	Pearson Correlation	.216	-.212	-.004	1	-.269	-.193	-.221	-.229
	Sig. (2-tailed)	.361	.369	.986		.251	.414	.349	.331

	N	20	20	20	20	20	20	20	20
LDRt	Pearson Correlation	-.677**	-.135	-.146	-.269	1	.543*	.548*	.545*
	Sig. (2-tailed)	.001	.571	.540	.251		.013	.012	.013
	N	20	20	20	20	20	20	20	20
Do	Pearson Correlation	-.295	-.037	-.145	-.193	.543*	1	.996**	.985**
	Sig. (2-tailed)	.207	.876	.541	.414	.013		.000	.000
	N	20	20	20	20	20	20	20	20
D1	Pearson Correlation	-.306	-.039	-.177	-.221	.548*	.996**	1	.996**
	Sig. (2-tailed)	.190	.870	.455	.349	.012	.000		.000
	N	20	20	20	20	20	20	20	20
D2	Pearson Correlation	-.307	-.041	-.210	-.229	.545*	.985**	.996**	1
	Sig. (2-tailed)	.187	.865	.374	.331	.013	.000	.000	
	N	20	20	20	20	20	20	20	20

**. Correlation is significant at the 0.01 level (2-tailed).

*. Correlation is significant at the 0.05 level (2-tailed).

Table 2. Pearson product moment correlations between significant variables

		Flicker ImplT	Big6avrC	LDRt	Do	D1
Flicker ImplT	Pearson Correlation	1	.749**	.475*	.644**	.670**
	Sig. (2-tailed)		.000	.034	.002	.001
	N	20	20	20	20	20
Big6avrC	Pearson Correlation	.749**	1	.475*	.725**	.751**
	Sig. (2-tailed)	.000		.034	.000	.000
	N	20	20	20	20	20
LDRt	Pearson Correlation	.475*	.475*	1	.543*	.548*
	Sig. (2-tailed)	.034	.034		.013	.012
	N	20	20	20	20	20
Do	Pearson Correlation	.644**	.725**	.543*	1	.996**
	Sig. (2-tailed)	.002	.000	.013		.000
	N	20	20	20	20	20
D1	Pearson Correlation	.670**	.751**	.548*	.996**	1
	Sig. (2-tailed)	.001	.000	.012	.000	

	N	20	20	20	20	20
D2	Pearson Correlation	.690**	.772**	.545*	.985**	.996**
	Sig. (2-tailed)	.001	.000	.013	.000	.000
	N	20	20	20	20	20

Correlations

		D2
Flicker ImplT	Pearson Correlation	.690**
	Sig. (2-tailed)	.001
	N	20
Big6avrC	Pearson Correlation	.772**
	Sig. (2-tailed)	.000
	N	20
LDRt	Pearson Correlation	.545*
	Sig. (2-tailed)	.013
	N	20
Do	Pearson Correlation	.985**
	Sig. (2-tailed)	.000
	N	20
D1	Pearson Correlation	.996**
	Sig. (2-tailed)	.000
	N	20
D2	Pearson Correlation	1
	Sig. (2-tailed)	
	N	20

**. Correlation is significant at the 0.01 level (2-tailed).

*. Correlation is significant at the 0.05 level (2-tailed).

Table 3. Partial correlation between Flicker ImpIT vs Big6avrc while controlling for LDRt, D_0, D_1, and D_2.

Control Variables			Flicker ImplT	Big6avrC
LDRt & Do & D1 & D2	Flicker ImplT	Correlation	1.000	.394
		Significance (2-tailed)	.	.131
		df	0	14
	Big6avrC	Correlation	.394	1.000
		Significance (2-tailed)	.131	.
		df	14	0

Table 4. Partial correlation between Flicker ImpIT vs LDRt while controlling for Big6avrc, D_0, D_1, and D_2.

Control Variables			Flicker ImpIT	LDRt
Do & D1 & D2 & Big6avrC	Flicker ImpIT	Correlation	1.000	.152
		Significance (2-tailed)	.	.574
		df	0	14
	LDRt	Correlation	.152	1.000
		Significance (2-tailed)	.574	.
		df	14	0

Table 5. Partial correlation between Flicker ImpIT vs D_0 while controlling for Big6avrc, LDRt, D_1, and D_2.

Control Variables			Flicker ImpIT	Do
D1 & D2 & Big6avrC & LDRt	Flicker ImpIT	Correlation	1.000	.005
		Significance (2-tailed)	.	.985
		df	0	14
	Do	Correlation	.005	1.000
		Significance (2-tailed)	.985	.
		df	14	0

Table 6. Partial correlation between Flicker ImpIT vs D_1 while controlling for Big6avrc, LDRt, D_0, and D_2.

Control Variables			Flicker ImpIT	D1
D2 & Big6avrC & LDRt & Do	Flicker ImpIT	Correlation	1.000	-.045
		Significance (2-tailed)	.	.867
		df	0	14
	D1	Correlation	-.045	1.000
		Significance (2-tailed)	.867	.
		df	14	0

Table 7. Partial correlation between Flicker ImpIT vs D_2 while controlling for Big6avrc, LDRt, D_1, and D_2.

Control Variables			Flicker ImpIT	D2
Big6avrC & LDRt & Do & D1	Flicker ImpIT	Correlation	1.000	.091
		Significance (2-tailed)	.	.736
		df	0	14
	D2	Correlation	.091	1.000
		Significance (2-tailed)	.736	.
		df	14	0
